# Validating potent anti-inflammatory and anti-rheumatoid properties of *Drynaria quercifolia* rhizome methanolic extract through in vitro*,* in vivo*,* in silico and GC-MS-based profiling

**DOI:** 10.1186/s12906-021-03265-7

**Published:** 2021-03-12

**Authors:** Debabrata Modak, Subhashis Paul, Sourav Sarkar, Subarna Thakur, Soumen Bhattacharjee

**Affiliations:** 1 Cell and Molecular Biology Laboratory, Department of Zoology, University of North Bengal, Raja Rammohunpur, Darjeeling, West Bengal 734013 India; 2 Department of Bioinformatics, University of North Bengal, Darjeeling, West Bengal 734013 India

**Keywords:** *Drynaria quercifolia*, Rheumatoid arthritis, Inflammation, Paw edema, GC-MS, Molecular docking

## Abstract

**Background:**

The fronds of *Drynaria quercifolia* have traditionally been used in rheumatic pain management. The goal of the present study was to validate the potent anti-inflammatory and anti-rheumatoid properties of the methanolic-extract of its rhizome using in vitro, in vivo and in silico strategies.

**Methods:**

The plant was collected and the methanolic extract was prepared from its rhizome. Protein denaturation test, hypotonicity and heat-induced haemolysis assays were performed in vitro. The in vivo anti-rheumatoid potential was assessed in Freund’s complete adjuvant (FCA)-induced Wistar rat model through inflammatory paw-edema, haematological, biochemical, radiological and histopathological measurements. Moreover, metabolites of methanolic extract were screened by gas chromatography-mass spectrometry (GC-MS) and 3D molecular structures of active components were utilized for in silico docking study using AutoDock.

**Results:**

In vitro results evinced a significant (*p* < 0.05) anti-inflammatory activity of the rhizome methanolic extract in a dose-linear response. Further, *Drynaria quercifolia* rhizome methanolic extract (DME) significantly ameliorated rheumatoid arthritis as indicated by the inhibition of arthritic paw-edema (in millimeter) in the rat rheumatoid arthritis models in both the low (57.71 ± 0.99, *p* < 0.01) and high dose groups (54.45 ± 1.30, *p* < 0.001) when compared to arthritic control. Treatment with DME also normalized the haematological (RBC, WBC, platelet counts and hemoglobin contents) and biochemical parameters (total protein, albumin, creatinine and ceruloplasmin) significantly (*p* < 0.05), which were further supported by histopathological and radiological analyses. Furthermore, GC-MS analysis of DME demonstrated the presence of 47 phytochemical compounds. Compounds like Squalene, Gamma Tocopherol, n-Hexadecanoic acid showed potent inhibition of cyclooxygenase-2 (COX-2), tumor necrosis factor (TNF-α), and interleukin (IL-6) in the docking analysis.

**Conclusion:**

Results from in vivo and in vitro studies indicated that DME possesses a potent anti-inflammatory and anti-arthritic activity. In silico studies delineated the emergent potent inhibitory effects of several bio-active components on the target inflammatory markers (COX-2, TNF-α and IL-6).

**Supplementary Information:**

The online version contains supplementary material available at 10.1186/s12906-021-03265-7.

## Background

Inflammation, the body’s defense mechanism, is a common biological cascade in response to foreign pathogens, tissue injury, or chemical irritation that acts by removing the injurious stimuli and begins the healing process which is rapid and self-limiting [1]. Both infectious and non-infectious stimuli can trigger an inflammatory pathway or acute inflammatory response that contributes to restoring physiological homeostasis. However, when prolonged, it may become chronic, enhance tissue inflammation and contribute to a variety of chronic inflammatory diseases like rheumatoid arthritis (RA) [2, 3]. RA is a chronic systemic autoimmune inflammatory disorder that has shown a worldwide prevalence of approximately 0.5 to 1% among adult individuals [4].

The disease RA is characterized by synovial hyperplasia, inflammatory cell infiltration in the synovial tissues and progression of this disease leads to the destruction of cartilages and bones and chronic disability of the patient [5]. Bone joint destruction is associated with chronic inflammation combined with the destruction of the surface and extracellular matrix of articular cartilage, and erosion of bone and if undiagnosed, it hampers the quality of lifestyle [6, 7]. The inflammation during RA results in the redness and swelling of the joint regions which is associated with the elevation of the temperature in the inflamed region and evokes severe pain [8]. The destruction of bones is mediated by the osteoclasts and the cartilage is mainly degraded by the matrix metalloproteinases in the inflammatory arthritic condition [9]. The cytokine including TNF-α, IL-6 and the COX-2 play vital role in the process. TNF-α and IL-6 are two apex cytokines contributing to the progression of inflammation whereas, COX-2 elevates the inflammatory pain through prostaglandin production [10]. Presently, the available treatment protocol involves the use of conventional corticosteroids, disease-modifying anti-rheumatic drugs (DMARDs), such as methotrexate, hydroxychloroquine; non-steroidal anti-rheumatic drugs (NSAIDs), such as indomethacin, ibuprofen, aspirin, etc. and immunosuppressive agents [11–13]. However, profound adverse effects are associated with the use of these agents, often leading to nephrotoxicity, gastrointestinal tract irritation, and hematological abnormalities and increased risk for cardiovascular diseases [14, 15]. Infliximab, a potent TNF-α inhibitor [16] and tocilizumab, a IL-6 inhibitor [17], have been used in the therapy against RA which are either less explored or have shown less diverse side-effects in the patients. Due to the persistent symptoms, adverse side-effects and the cost involved in the current treatment protocol, alternative strategies are drawing more attention nowadays.

In the last few decades, the search for a safe alternative remedy from herbal resources has been an area of interest as herbs play a key role in the modern medication system and constitute a major reservoir for potentially active medicinal compounds all over the World. Medicinal plants have been proven to be potent and efficacious in ameliorating RA [9]. The field of RA research has progressed rapidly towards herbal treatment, as they are effective in ameliorating the pain and inflammation associated with the disease, have lesser side effects and also at a low cost. *Drynaria quercifolia* (commonly called ‘Pankhiraj’ in Bengali/India) has been one of the pioneer medicinal plants which have a great medicinal value against various diseases. It can be either epiphytic (when growing on trees) or epipetric (when growing on rocks) in nature. *Drynaria quercifolia* belongs to the family *Polypodiaceae* of Pteridophyta and is widely distributed in tropical and subtropical countries like India, Bangladesh, Pakistan, North America, and Africa [18, 19]. Traditionally, the fronds of this plant are reported to be used by different tribal communities of India in the treatment of various diseases like typhoid fever, chronic jaundice, headache, cough, cholera and skin disease [19–21]. The soup prepared from the rhizomes of *Drynaria quercifolia* is popularly used by some tribes of Eastern Ghats, Tamil Nadu to get relief from rheumatic complaints [22]. Consumption of *Drynaria quercifolia* can help to heal and strengthen broken bones and also be used to promote the treatment of bone fracture by the sub-Himalayan tribal communities like Mech, Toto, Rabha and also Koch tribe from the Cooch Behar region of West Bengal, India [23, 24]. The fronds have a stringent property and are found to strengthen and promote the repair of sinews, muscles, and bones [25]. Phytochemical screening of *Drynaria quercifolia* has confirmed the presence of flavonoids, alkaloids, saponin, tannins, and other chemical substances [26]. The efficacy of the *Drynaria quercifolia* rhizome has already been shown in different in vitro and in vivo models with respect to antimicrobial [27], analgesic [19], anti-inflammatory [28] and hepatoprotective activities [29]. Membrane-stabilization and thrombolytic potentials of the plant has also been reported [30]. The rhizome of this plant has also shown its efficacy by decreasing lysosomal enzymes levels, protein bound carbohydrate levels, urinary degradative collagen levels and serum cytokines level in inflammatory arthritis model [31]. All the initial studies have drawn significant evidences regarding the medicinal properties of the plant rhizome extract.

The current study was aimed at validating the traditional uses of *Drynaria quercifolia* rhizome extracts in inflammatory conditions through in vitro and in vivo studies. The present study also intended to identify the active constituents of methanolic extract using GC-MS analysis and to identify potential active anti-inflammatory components against COX-2, TNF-α and IL-6, key players in the inflammatory pathway, through in silico molecular docking studies.

## Materials and method

### Collection of rhizome of *D. quercifolia*

Rhizomes of naturally growing *Drynaria quercifolia (L.)* J. Sm. (Class Polypodiopsida, Order Polypodiales, and Family *Polypodiaceae*) were collected from the North Bengal University Campus during September 2018. Collected plant specimens were authenticated by Taxonomy of Angiosperm and Biosystematics Laboratory, Department of Botany, University of North Bengal. A voucher specimen was deposited with the accession number: 09746 at the herbarium of the Department of Botany, University of North Bengal. A flow sheet of methodology has been provided as an additional file (see Additional file 1).

### Preparation of methanolic extract of *D. quercifolia*

Fresh rhizomes (~ 10 kg) of *Drynaria quercifolia (L.)* J. Sm. were properly cleaned and washed with tap water to remove the dust particles. Then the wooly brown scales of the rhizomes were removed and the clean rhizomes were shade dried at room temperature for 2 weeks to obtain about 760 g dry sample. The dried rhizomes were then crushed into powder using an electrical grinder. The coarsely powdered rhizomes (30 g) were successfully extracted with 100 ml of HPLC grade methanol (Merck, India) using Soxhlet apparatus for 6–7 h. The obtained extract was then concentrated using a Buchi type rotary evaporator (Cole Parmer RV1010D596, India) under reduced pressure and temperature (45 °C) and the percentage yield of the extract was 7% (w/w). The resultant *Drynaria quercifolia* rhizome methanolic extract (DME) was stored in an air tight container at 4 °C for further use. For animal feeding, DME was reconstituted in 0.5% dimethyl sulfoxide (DMSO) (Sigma-Aldrich, USA).

### In vitro anti-inflammatory assays

#### Protein denaturation test

The test was performed as described by previous protocols [32, 33]. Chicken egg albumin was used as a protein source and the experiment was carried out in triplicate sets (per dose). Briefly, the reaction mixture (5 ml) consisted of 0.2 ml of egg albumin, 2.8 ml of phosphate-buffered saline (PBS, pH 6.4) and 2 ml of varying concentrations of DME resulting in the final concentrations of 600, 800, and 1000 μg/ml. The negative control group had 2 ml of distilled water instead of DME. Diclofenac sodium (Novartis India Ltd., India) at the final concentration (100 μg/ml) was used as a standard drug. The reaction mixture was then incubated at 37 °C for 15 min and then the mixture was heated at 70 °C for 15 min in a regulated water bath. After cooling, the turbidity of the reaction mixture was measured at 660 nm. The percentage inhibition (PI) of protein denaturation was calculated using the following formula [32, 33]:

$$ \mathrm{PI}=\frac{\left(\mathrm{OD}2-\mathrm{OD}1\right)}{\mathrm{OD}2}\times 100 $$

Where, OD1 = Absorbance of heated test sample;

OD2 = Absorbance of heated negative control sample

### Membrane stabilization activity

#### Preparation of erythrocyte suspension

Erythrocyte suspension was prepared according to previously described protocol [33, 34]. Briefly, 3 ml of whole blood was collected from a healthy volunteer in a tube containing ethylene-diamine-tetraacetic acid (EDTA), centrifuged at 3000 rpm for 10 min, and washed three times with an equal volume of normal saline (0.9%). Then the volume of the dissolved red blood pellets was measured and reconstituted as a 40% (v/v) suspension with an isotonic buffer solution (10 mM sodium phosphate buffer, pH 7.4).

#### Hypotonic solution induced haemolysis

For this test, the hypotonic solution (distilled water) (5 ml) containing 600, 800, and 1000 μg/ml of DME was put in 3 pairs (per dose) on centrifuge tubes. In another set of centrifuge tubes, isotonic buffer solution (5 ml) containing the similar graded doses of DME were taken in 3 pairs (per dose). Negative control tubes contained 5 ml of hypotonic solution (distilled water) and indomethacin (200 μg/ml) (Cipla, India) served as the standard drug. Stock erythrocyte suspension (100 μl) was added to each tube, and after gentle mixing, the tubes were incubated at 37°C for 1 h. After incubation, the reaction mixture was centrifuged for 3 min at 1300 g at room temperature and the absorbance (OD) of the supernatant was measured at 540 nm. The percentage inhibition (PI) of haemolysis was calculated using the following equation [33, 34]:

$$ \mathrm{PI}=\left[1-\left(\frac{\mathrm{OD}2-\mathrm{OD}1}{\mathrm{OD}3-\mathrm{OD}1}\right)\right]\times 100 $$

Where,

OD1 = Absorbance of test sample in isotonic solution

OD2 = Absorbance of test sample in hypotonic solution (distilled water)

OD3 = Absorbance of negative control sample in hypotonic solution (distilled water)

#### Heat induced haemolysis

For this test, the isotonic buffer solution (5 ml) containing 600, 800, and 1000 μg/ml of DME were assorted in 3 sets (per dose) of centrifuge tubes. Control tubes contained 5 ml of the vehicle (distilled water) and the standard dose group contained 5 ml isotonic buffer solution containing indomethacin (200 μg/ml). Erythrocyte suspension (100 μl) was added to each tube and gently mixed. Then the tubes were incubated at 54 °C for 20 min in a regulated water bath. At the end of the incubation, the reaction mixtures were centrifuged at 1300 g for 3 min at room temperature and the absorbance (OD) of the supernatant was measured at 540 nm. The percent inhibition (PI) of haemolysis was calculated using the following equation [33]:

$$ \mathrm{PI}=\frac{\left(\mathrm{OD}2-\mathrm{OD}1\right)}{\mathrm{OD}2}\times 100 $$

Where,

OD1 = Absorbance of heated test sample (isotonic buffer).

OD2 = Absorbance of heated negative control sample (distilled water).

### In vivo anti-inflammatory study

#### Animal maintenance

Wistar albino rats of both sexes (8–12 weeks old, 120 ± 10 g) were purchased from authorized animal dealers (Chakraborty Enterprise, Kolkata, India; Regd. No. 1443/PO/Bt/s/11/CPCSEA). All animals were housed under well-ventilated polypropylene cages (Tarsons, India) with paddy husk as bedding material. All the experimental rats were maintained in the animal house of the Department of Zoology, University of North Bengal with a controlled environmental condition maintained at a constant room temperature (25 ± 3 °C) and under a constant 12-h dark/light cycle. Rats were provided with sufficient food and water ad libitum. The study has been approved by the Institutional Animal Ethical Committee (IAEC) of CPCSEA (Committee for the Purpose of Control and Supervision of Experiments on Animals) of the University of North Bengal, West Bengal, India (IAEC/NBU/2018/03). Animals were acclimatized for 2 weeks before the study.

#### Acute oral toxicity test

Acute toxicity test was performed according to the Organization for Economic Cooperation and Development guidelines (OECD) 423 [35]. In the acute toxicity tests, Wistar albino rats of both sexes were clustered into five groups; each contained 6 rats; 3 males and 3 females. The first group contained normal animals; the other four were considered as experimental groups where DME was administrated orally by using gavage in single doses of 250 mg/kg b.w., 500 mg/kg b.w., 1000 mg/kg b.w. and 2000 mg/kg b.w. The general behaviors, such as aggressiveness, the consumption rate of food and water intake, sedation, diarrhea, rising of fur, lethargy were continuously observed individually during the first 30 mins and periodically for 24 h after the treatments were administered. All animals were observed individually and special attention was given during the first 4 h daily and thereafter, for a period of 14 days for any late sign of toxicological effects.

### Experimental design for anti-arthritic property of DME in FCA-induced inflammatory arthritis model

#### Experimental groups

A total number of twenty-four (24) Wistar albino male rats (120 ± 10 g) randomly divided into four groups of six rats each as under:

- Group 1 (Arthritic control): Arthritic rats received orally normal saline (p.o.) only, from day 0 to day 28.
- Group 2 (Low dose group of DME): Rats were treated with DME (250 mg/kg b.w.). The therapy was commenced on day 0 and continued till the 28th day.
- Group 3 (High dose group of DME): Rats were treated with DME (500 mg/kg b.w.). The therapy was commenced on day 0 and continued till the 28th day.
- Group 4 (Vehicle control): 0.5% of DMSO was administered (p.o.) to each healthy rat of the vehicle group. The duration of treatment was similar to other experimental groups.

#### Induction of arthritis

The adjuvant-induced RA model performed in this study was similar to that described by previously published protocol [36]. Arthritis was induced by intradermal injection of 0.1 ml of FCA into the sub-plantar region of the right hind paws of the rats. On day 0, FCA (Sigma-Aldrich, India) was injected into all the animals in groups, except to the vehicle control group animals. The animals received a booster dose of 0.1 ml of FCA on the 14th day. The treatment commenced on day 0 of arthritic induction and continued till the 28th day. DME doses were given once each day according to experimental groups and all animals were sacrificed on day 28.

#### Assessment of paw edema

The severity of arthritis was blindly measured by measuring the paw edema with the help of a vernier caliper. Paw circumferences were measured at regular intervals of 2 to 3 days from the day of arthritis induction, using the formula 2π√[(A^2^ + B^2^)/2], where A and B were the measures of paw circumferences at two different axis lengths each placed at right angle to the other; one at dorsal-plantar side and the other at right-left side [36]. To minimize the error rate, the mean values of three measurements were taken for each animal.

#### Haematological parameters

After the treatment schedule, all animals were anesthetized with sodium pentobarbital (60 mg/kg; i.p.) and euthanized by cervical decapitation. Thereafter, blood samples were obtained through the cardiac puncture into EDTA coated vials and different hematological parameters (RBC count, WBC count, platelet count, and Hb content) were determined using an automated haematology analyzer (Sysmex XN-1000, Mumbai, India).

#### Biochemical parameters

For serum analyses, blood samples were centrifuged (5000 rpm for 10 min) and serums were collected. Total protein, creatinine and albumin were estimated by Coral Kits (Coral clinical systems, India) following the manufactures’ protocol. Serum ceruloplasmin was evaluated by p-phenylenediamine oxidase activity [37].

#### Histopathological parameters

The ankle joints were surgically removed and placed in 10% formalin for fixation. A solution of 3% HCl was used for decalcification [36]. The old solution was replaced with a fresh solution every 5 days till optimal decalcification. Liver and kidney samples were also collected and fixed in 4% formalin. In the next step, the tissues were dehydrated using serial dilutions of ethanol and subsequently embedded in paraffin wax. Tissues were cut into longitudinal sections (for ankle joint) and transverse sections (for liver and kidney) at 5 μm thickness and stained with haematoxylin and eosin. Thereafter, microscopic examinations were done under a light microscope (Nikon Eclipse E200, Nikon, Tokyo, Japan) with 10X and 40X objectives [36] and respectively scale bar has been attached (100 μm and 25 μm).

#### Radiological parameters

High-resolution digital X-ray imaging of right hind-limbs was performed blind-folded to assess the severity of FCA induced arthritis. X-ray imaging was performed in a fixed X-ray machine (Allengers 325/625, Mumbai, India) at 50 kV peak, 50 mA and the exposure time was 3 seconds. The limb region images were manually cropped from the digitized image and were analyzed [38].

#### GC-MS study

DME extract was subjected to GC-MS analysis using GCMS-QP2010 Ultra (Shimadzu, Japan) attached with a fused silica capillary column Rtx-5MS (column length 30.0 m and column diameter 0.25 mm) at AIRF center, JNU, New Delhi following standard protocols [39, 40]. The analysis was performed by manually injecting 1 μl of the sample with a split ratio of 10.0. Pure helium gas (99.9%) was used as the carrier gas at a constant flow rate of 1.21 ml/min. The injector was operated at 260 °C and the ion source temperature was set at 230 °C. The separation of the nonpolar components was achieved using a temperature program of 60 °C for 3 min, then ramped at 10 °C/min to 280 °C and held for 25 min. The mass spectra were taken at a scan-interval of 0.2 seconds and fragments range was scanned from 40 to 650 m/z. The total run time of the program was 50 min. The relative quantity of the chemical components present in DME was evaluated using total ion count (TIC) and expressed as a percentage based on peak area produced in the chromatogram. The spectrums of the component were identified based on the comparison of their mass spectra with those available in the computer library (NIST11 and Willey 8) attached to the GC-MS instrument [39, 40].

#### Molecular docking studies

Phytochemicals identified in GC-MS analysis were selected for molecular docking studies. The structures of selected phytochemicals (in. SDF format) were obtained from the NCBI PubChem database [41]. SMILES server was utilized for converting the. SDF to PDB file format [42] and these structures was then served as the ligand. The crystal structure of COX-2 (PDB id- 4COX), TNF-α (PDB id- 2AZ5), and IL-6 (PDB id- 1ALU) were obtained from protein data bank [43] which were complexed with an inhibitor [44–46]. The resolution of protein structures were 2.9 Å, 2.10 Å, and 1.90 Å for COX-2, TNF-α and IL-6 respectively, which are sufficient for the docking study. The original ligand was removed before the docking study using Discovery Studio Visualizer. Then the target protein was fed into the CASTp server for predicting the active sites [47]. Water molecules in the protein structures were replaced by polar hydrogen and Kollman charges were added it. The AutoDock tool 1.5.6 was used for grid-based in silico molecular docking studies [48]. The grid-size of the receptor molecule was set at 60 × 60 × 60 and the rest parameters were left to default. The Lamarckian genetic algorithm (GA) method was used for docking analysis [49] and 100 GA runs were implemented for each ligand molecule. Both Autogrid 4 and Autodock 4 computations were performed on the Cygwin platform. The docking results were analyzed using Discovery Studio Visualizer software.

#### Statistical analysis

Quantitative data concerning the paw circumference measurement was expressed as mean ± standard Error Mean (S.E.M). For the remaining biochemical assays, the data are expressed as mean ± standard deviation (S.D). Comparison between more than two groups was done using one-way analysis of variance (ANOVA) or by two-way ANOVA following the post hoc analysis with a Dunnett’s multiple comparisons test. Inter-group variations between more than two groups were measured using ANOVA followed by Tukey’s post hoc test. Values of *p* ≤ 0.05 were taken to indicate a statistical difference. All the statistical analyses were performed using Graph Pad Prism Version 7.00 for Windows (GraphPad Software Inc., San Diego, USA).

## Results

### Results of in vitro anti-inflammatory assays

#### Effect of DME on protein denaturation test

**Fig. 1:**
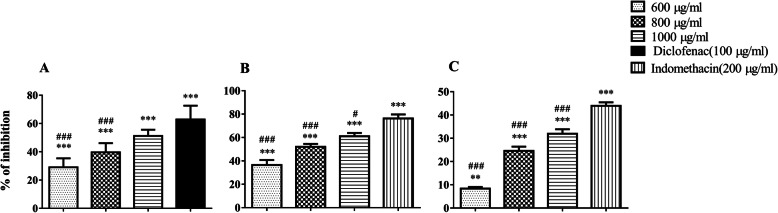
In vitro anti-inflammatory activities of DME. Treatment with DME significantly inhibited protein denaturation (**a**), hypotonicity-induced haemolysis (**b**) and heat-induced haemolysis (**c**) as compared to both the positive and negative control groups. The negative control group is considered to have 100% protein denaturation or hypotonicity or heat induced haemolysis (bar not shown in the graphs). * indicates the comparison between the dose groups and negative control group; # indicates the comparison between the dose groups and standard drug group

In the present study, DME showed good anti-inflammatory activity with a dose linear response when we compared with the negative control group (F = 58.5). DME showed mean inhibition of protein denaturation of 29 ± 6.34%, 39.6 ± 6.46% and 51.2 ± 4.41% for the doses of 600 μg/ml, 800 μg/ml and 1000 μg/ml respectively (Table 1). The ability of extract of DME to inhibit denaturation of protein was found to be statistically significant (*p* ≤ 0.05) when compared to the negative control dose group (distilled water). Diclofenac, the standard NSAID drug, showed the maximum inhibition, 62.8 ± 9.79% at a concentration of 100 μg/ml (Fig. 1a). The 1000 μg/ml dose group showed no difference in protein denaturation inhibitory activity when compared with the standard Diclofenac (F = 58.5). However, all the 600 μg/ml, 800 μg/ml and 1000 μg/ml dose groups showed dose dependent relationship for the protein denaturation inhibition, when compared with standard drug group (Fig. 1a).

**Table 1:** Inhibition properties of DME in protein denaturation and membrane stabilization (both hypotonicity and heat induced) as compared to negative control group

Test samples	Concentration (μg/ml)	Mean% of inhibition of haemolysis ± S.D		
		Protein denaturation test	Hypotonicity induced	Heat induced
DME	600	29 ± 6.34 ***	36.5 ± 7.3***	8.37 ± 1.24**
	800	39.6 ± 6.46 ***	52.1 ± 4.22***	24.6 ± 3.21***
	1000	51.2 ± 4.41***	61.1 ± 4.81***	31.9 ± 3.35***
^a^ Diclofenac	100	62.8 ± 9.79***	–	–
^b^ Indomethacin	200	–	76.3 ± 5.82***	43.9 ± 3.03***

***indicates *p* ≤ 0.001, **indicates *p* ≤ 0.01, and *indicates *p* ≤ 0.05

^a^ Diclofenac served as a standard drug for anti-protein denaturation agents

^b^ Indomethacin served standard drug for a membrane stabilizing activity

#### Effect of DME on hypotonicity induced haemolysis

The DME protected the erythrocyte in hypotonicity induced haemolysis in a concentration-related manner. Within experimental groups, the maximum protective effect was seen in 1000 μg/ml DME dose group (61.1 ± 4.81%). Results showed that the DME dose groups (600 μg/ml, 800 μg/ml and 1000 μg/ml) significantly (F = 58.5; *p* ≤ 0.05) inhibited the haemolysis in hypotonic conditions (Fig. 1b). However, the percentage of inhibitions of haemolysis shown by the DME doses were lower than that obtained for standard NSAID drug, indomethacin (76.3 ± 5.82%) (Table 1). When we compared all the dose groups of DME with the standard drug group, a significant dose-dependent haemolysis was observed (Fig. 1b).

#### Effect of DME on heat induced haemolysis

All the doses of DME (600 μg/ml, 800 μg/ml and 1000 μg/ml) effectively inhibited the heat induced haemolysis (Table 1). Within the experimental groups, maximum inhibition (31.9 ± 3.35%) was observed in the 1000 μg/ml dose group. The percentage of inhibition of haemolysis caused by DME in all experimental groups was found to be dose-dependent and statistically significant (F = 193; *p* ≤ 0.05). However, the standard drug, indomethacin showed the highest protection (43.9 ± 3.03%) at a concentration of 200 μg/ml (Fig. 1c). When we compared with standard-drug group, all doses of DME (600 μg/ml, 800 μg/ml and 1000 μg/ml) significantly (*p* ≤ 0.05) inhibited the denatured protein.

### Result of DME *on* in vivo anti-inflammatory study

#### Acute oral toxicity test of DME

All rats treated with different concentrations of DME were alive for all 14 days of observation. The DME did not show any mortality or behavioral abnormalities in the immediate 24 h after DME feeding as well as in the subsequent 14 days post-treatment. Normal body weight gain was observed in all animal groups (data not shown). Hence, the DME was considered non-toxic up to a dose of 2000 mg/kg body weight.

#### Effect of DME on FCA induced paw edema

**Fig. 2:**
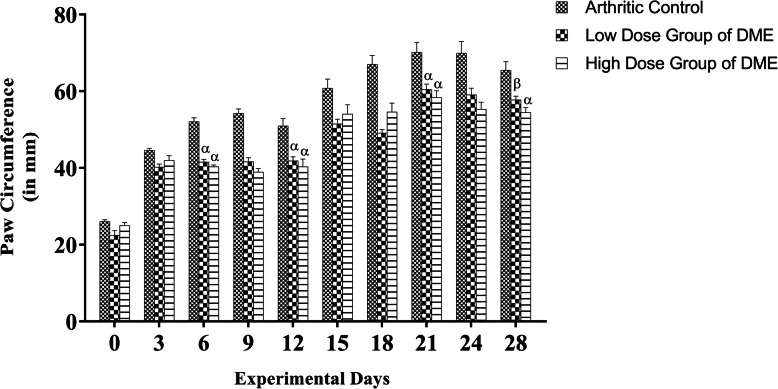
The data showed the comparisons of rat paw-edema expressed in circumference (mm) in different experimental groups. Treatment with DME in both low and high dose group significantly ameliorated paw-edema during the experimental schedule, when compared with arthritic control group animals. Vehicle groups’ data is not shown in bar diagram. The data is expressed as mean ± SEM of six animals. Two-way ANOVA was performed followed by Dunnett’s multiple comparisons test. α indicates *p* ≤ 0.001 and β indicates *p* ≤ 0.01

There was a significant increase (*p* < 0.05) of paw-circumference (in mm) in the arthritic control group when compared with the DME-treated experimental groups from the 6th day onwards after the FCA injection; however, no significant inter-group difference was seen on the 3rd day (Fig. 2). On day 12th, before the booster dose of FCA was given, both the low dose (41.82 ± 1.09; *p* < 0.001) and high dose (40.31 ± 2.02; *p* < 0.001) group of DME showed significant reduction of the paw swelling when compared to the arthritic control group (50.93 ± 1.92). Following the booster dose of FCA on 14th day, all the three groups achieved inflammatory swelling and no obvious significant inter-group difference in swelling was observed during 15th and 18th day of the experiment. The paw-swelling showed a significant reduction on the 21st day in both the low (60.44 ± 1.44; *p* < 0.001) and high dose groups (58.39 ± 1.72; *p* < 0.001) of DME when compared to the arthritic control group (70.13 ± 2.59). On the last day of anti-arthritic treatment, we found a significant (*p* < 0.001) inhibition of paw-swelling in high dose group of DME (54.45 ± 1.30) and a significant reduction (*p* < 0.01) of paw swelling in low dose group of DME (57.71 ± 0.99) when compared to control arthritic group (65.43 ± 2.31). However, the two experimental dose-groups showed no significant difference in their paw circumference measurements during the experimental tenure when compared with each other following Tukey’s post hoc test.

#### Assessment of haematological parameters

**Fig. 3:**
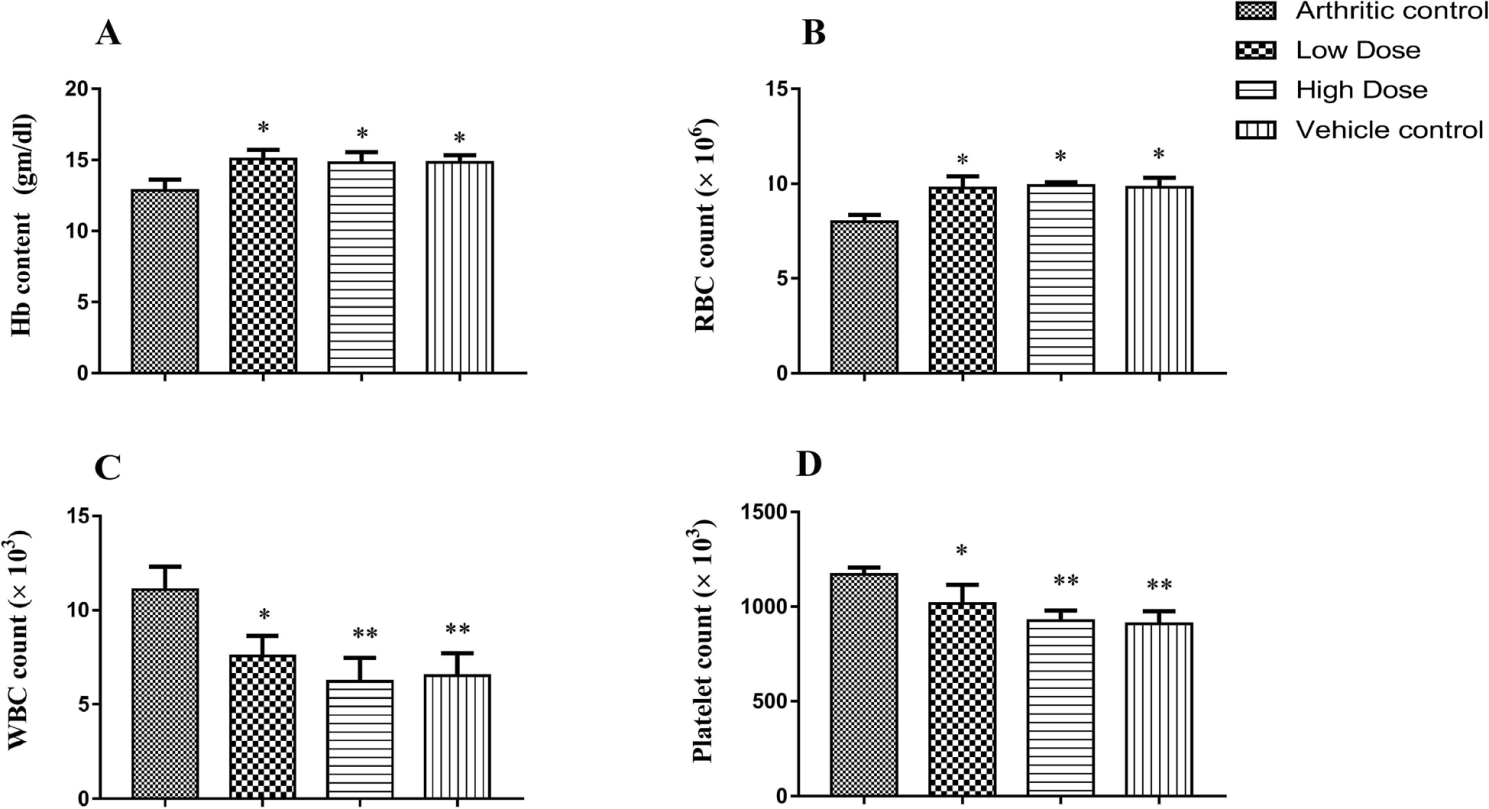
Haematological parameters of different experimental rat groups after treatment schedule. Treatment with DME nearly normalized the Hb content (**a**) and RBC (**b**) counts and also showed a significant decrease of elevated WBC (**c**) and platelet (**d**) counts when compared with arthritic control groups. All data represented as mean ± SD of six animals and analyzed by one-way ANOVA followed by Dunnett’s post hoc test compared with arthritic control group, where ** indicates *p* ≤ 0.01, and * indicates *p* ≤ 0.05

The haematological parameters including Hb content, total RBC, total WBC and total platelet count were measured and compared to the arthritic control group. Both the low dose (14.8 ± 0.75) and high dose (15.1 ± 0.66) treated DME group showed a significant (F = 4.71; *p* ≤ 0.05) increase of Hb content when compared with arthritic control (12.9 ± 0.77) (Fig. 3a). A similar significant (F = 6.75; *p* ≤ 0.05) normalization of RBC count was also found in both the low dose (9.78 ± 0.60) and high dose (9.9 ± 0.18) treated DME group as compared with the arthritic control group (7.99 ± 0.36) (Fig. 3b). We found a significant (F = 9.73) decrease in WBC count in both the low dose (7.57 ± 1.07; *p* ≤ 0.05) and high dose (6.2 ± 1.25; *p* ≤ 0.01) treated DME group when compared with arthritic control (11.1 ± 1.2) (Fig. 3c). Platelet count was also significantly decreased (F = 16.2) after treatment in the low dose (1017 ± 97.5; *p* ≤ 0.05) and high dose (926 ± 53; *p* ≤ 0.01) group of DME, as compared with arthritic control (1171 ± 36.2) (Fig. 3d).

#### Assessment of biochemical parameters

**Fig. 4:**
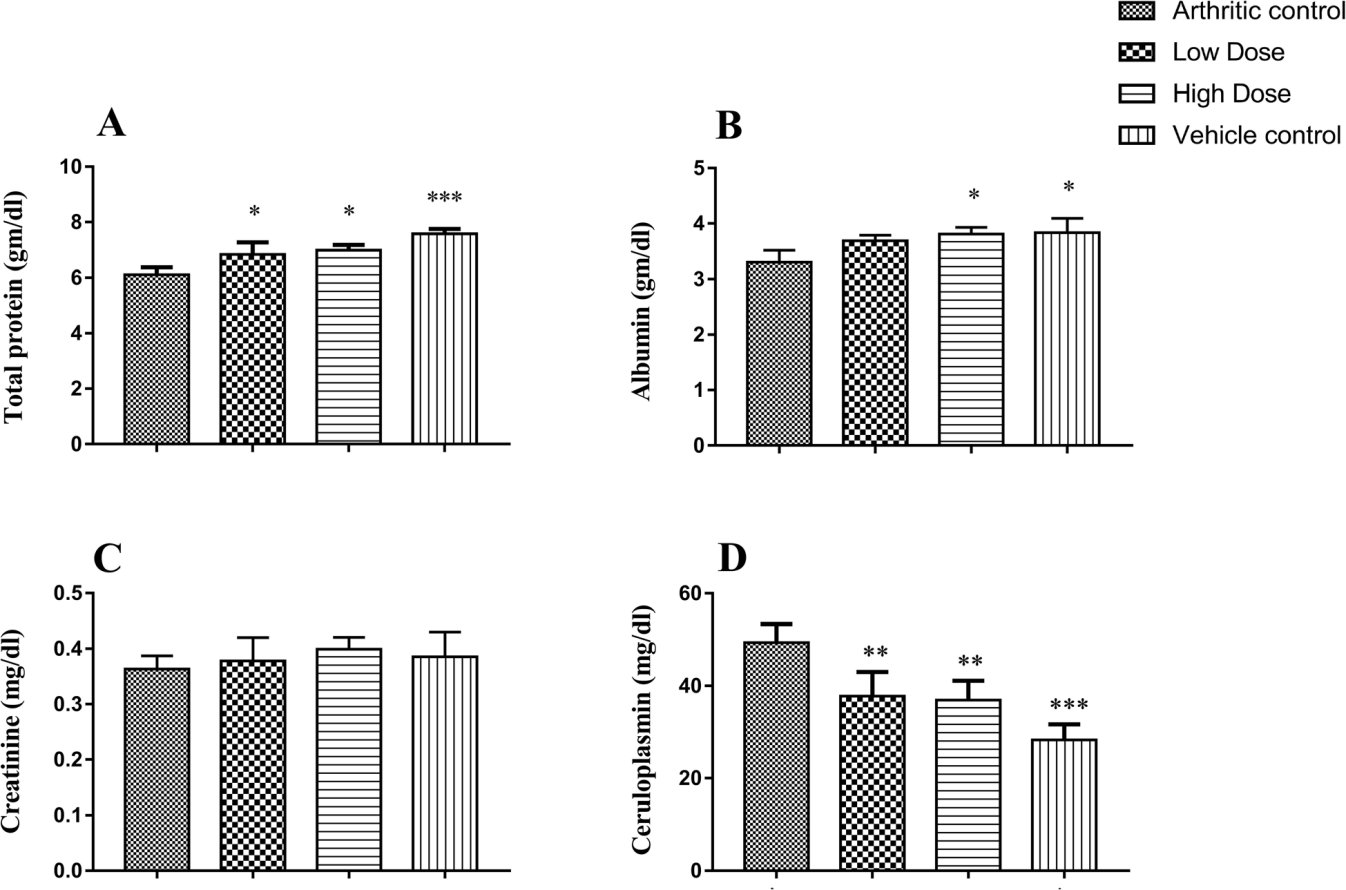
Biochemical parameters of different experimental rat groups after treatment schedule. Treatment with DME nearly normalized the total protein (**a**) and albumin levels (**b**); showed no alteration in creatinine level (**c**) and normalized the elevated serum ceruloplasmin level (**d**) when compared with arthritic control groups. All data represented as mean ± SD of six animals and analyzed by One-way ANOVA followed by Dunnett’s post hoc test where ***indicates *p* ≤ 0.001, **indicates *p* ≤ 0.01, and *indicates *p* ≤ 0.05

We found a significant (F = 13.4) increase of serum total protein level after the DME treatment. Both the low dose (6.84 ± 0.43) and high dose (6.98 ± 0.19) treated DME group showed a significant (*p* ≤ 0.05) increase of total protein level when compared with arthritic control (6.11 ± 0.25) (Fig. 4a). In the case of the serum albumin level, no significant (F = 5.3) difference was observed in the low dose treated group (3.69 ± 0.09) when compared with arthritic control (3.3 ± 0.21). However, the high dose group (3.81 ± 0.11) animals showed a significant (*p* ≤ 0.05) increase of serum albumin when compared with the arthritic control group (Fig. 4b). In case of creatinine level, DME did not produce any alteration in all the experimental groups, as we found a non-significant (F = 0.56) difference when compared with each other (Fig. 4c). Furthermore, a significant (F = 16.5) decrease in elevated serum ceruloplasmin was also observed in both the low dose (37.8 ± 5.23; *p* ≤ 0.01) and high dose (36.9 ± 4.17; *p* ≤ 0.01) treated DME group when compared with the arthritic control group (49.4 ± 4.03) (Fig. 4d).

#### Effect of DME on radiological and histopathological analyses

**Fig. 5:**
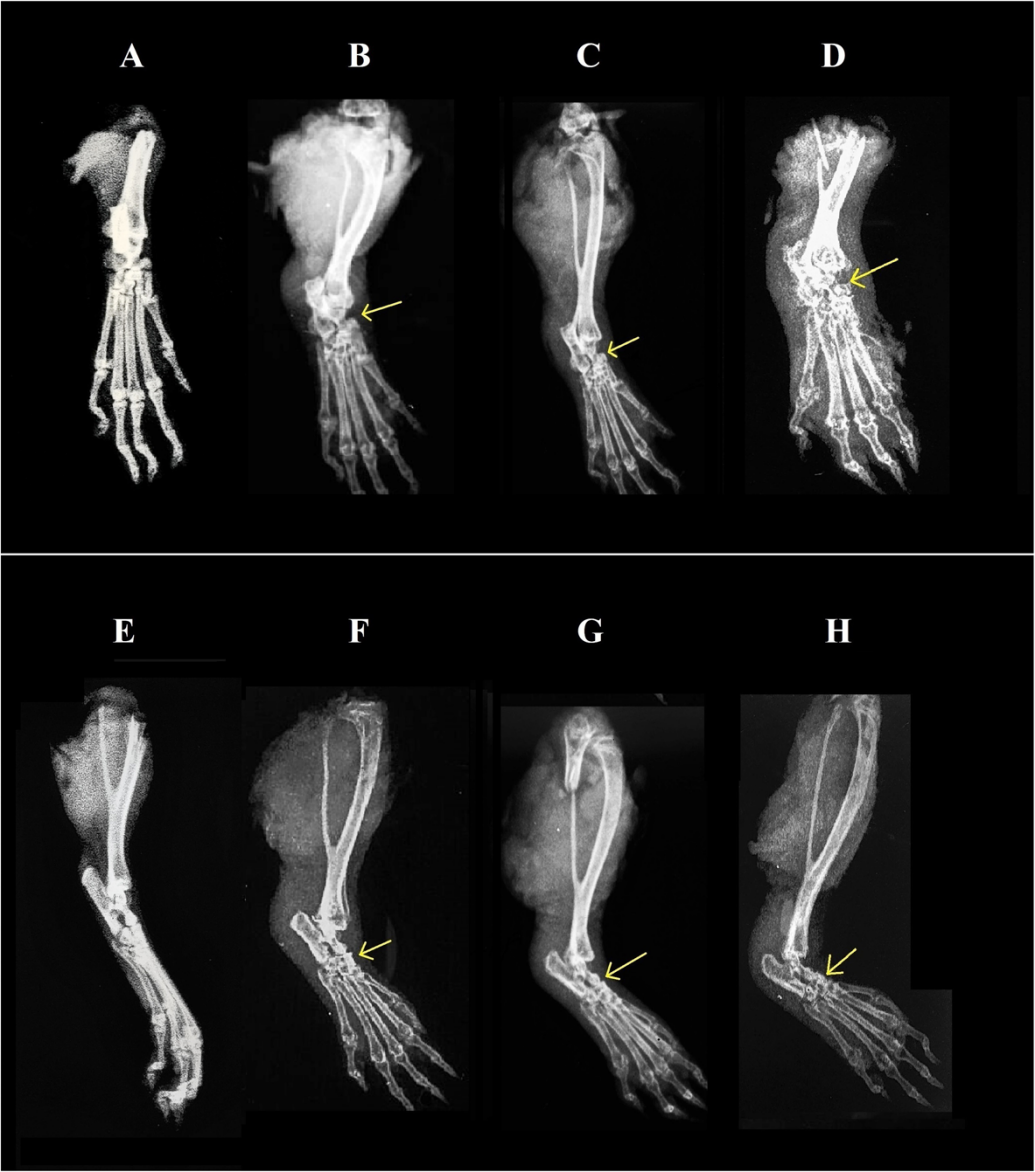
Radiological analyses of right hind paw ankle joints of experimental rats. Arrow marks indicate the swelling and degradation of cartilage in experimental animals. Upper panel (**a**, **b**, **c**, **d**) shows the horizontal view and lower panel (**e**, **f**, **g**, **h**) shows the angular view of the same bone joints; where, **a** and **e** = vehicle group, **b** and **f** = low dose group, **c** and **g** = high dose group and **d** and **h** = arthritic control group

In conventional radiographic imaging, radiographs of the right hind paws were taken at the termination of the experimental schedule and evaluated visually. It was evident that the arthritic control group (Fig. 5d and h) developed progressive soft-tissue swelling, irregular joint space and prominent bone erosion. However, in low (Fig. 5b and f) and high dose (Fig. 5c and g) groups, the joint space appeared to be normal and soft tissue swelling was decreased compared to arthritic control groups. In the vehicle control groups (Fig. 5a and e), a normal bone structure was observed.

**Fig. 6:**
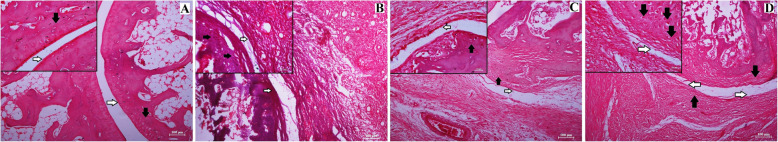
Histological sections of rat paw (ankle) joints obtained from different experimental groups. White arrows represent the destruction in cartilage lining and the black arrows represent the immune cell infiltration in all experimental groups. **a** = vehicle, **b** = low dose, **c** = high dose and **d** = arthritic control group. The main images represent sections observed under 10X magnification; inset represents 40X magnified images. Scale bars are 100 μm in 10X and 25 μm in 40X

The images obtained from the histological sections (longitudinal) of the arthritic joints of different groups differed significantly in their structures. In the image, the white arrows refer to the cartilage lining and black arrows refer to the infiltration of WBCs. The sections obtained from the vehicle group (Fig. 6a) showed smooth joint cartilage lining and no significant infiltration of immune cells was observed in the cartilage. In the arthritic control joint (Fig. 6d), contrastingly, irregular cartilage lining (white arrow) was observed along with the cartilage being highly infiltrated by the immune cells (black arrow) due to severe inflammatory arthritis. In the sections obtained from low dose (Fig. 6b) and high dose (Fig. 6c) groups, the number of infiltrating cells decreased in the cartilage region compared to the arthritic control group and the cartilage lining was restored to normalcy.

**Fig. 7:**
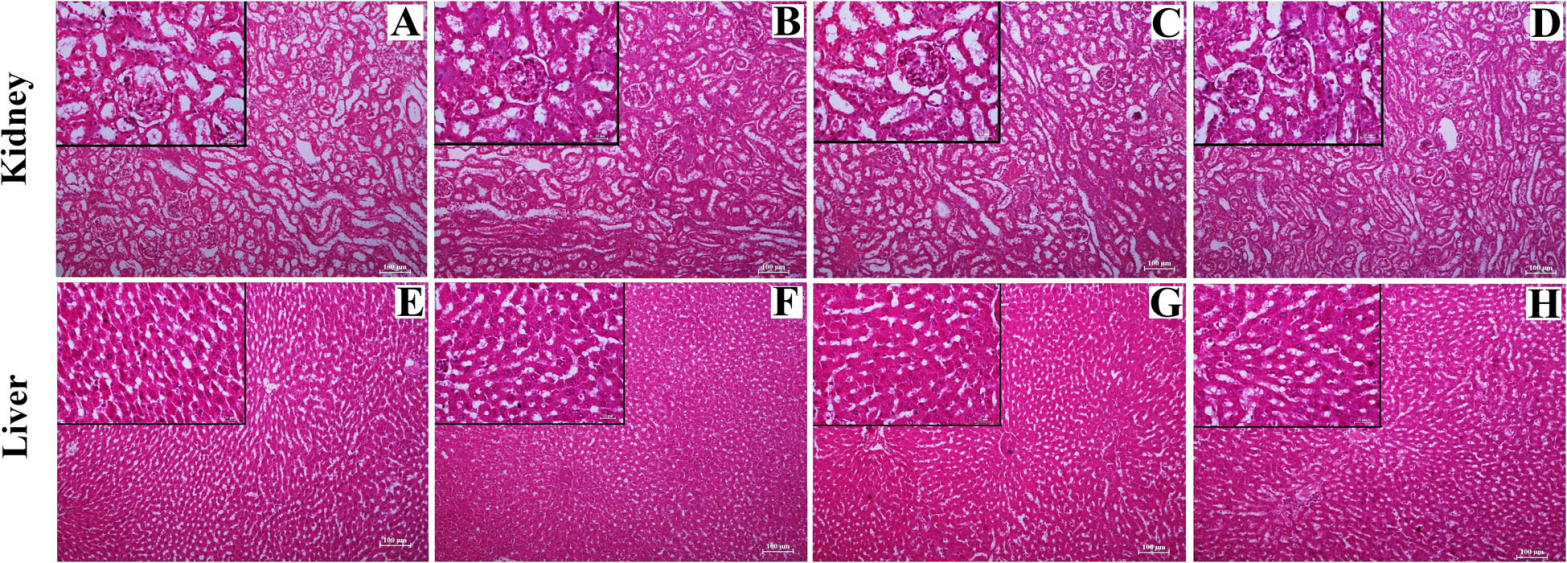
Histological sections of kidney (**a**, **b**, **c**, and **d**) and liver (**e**, **f**, **g** and **h**) obtained from different experimental groups. **a** and **e** = vehicle group, **b** and **f** = low dose group, **c** and **g** = high dose group and **d** and **h** = arthritic control group. The main images represent sections observed under 10X magnification; inset represents 40X magnified images. Scale bars are 100 μm in 10X and 25 μm in 40X

There were no observable structural changes in kidney and liver sections between the different experimental groups. The sections of kidneys (Fig. 7a-d, upper panel) showed well organized Bowman’s capsule cells as well as the organized lining of ducts. There was no significant structural change between groups. The liver sections (Fig. 7e-h, lower panel) of all the groups also showed well-organized cellular structure and no abnormalities were seen in any groups. A, E represent vehicle group; B, F represent low dose group; C, G represents high dose group and D, H represents arthritic control group.

#### GC-MS analysis of DME

**Fig. 8:**
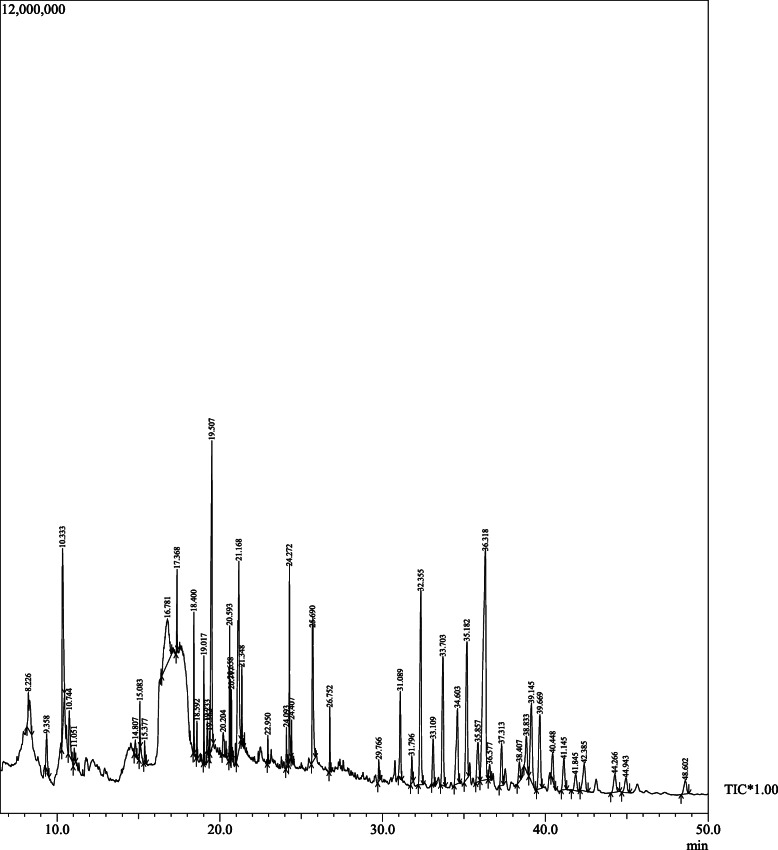
GC-MS chromatogram of methanolic rhizome extract of *Drynaria quercifolia* (see text for detail)

The DME was subjected to phytochemical analysis using optimized GC-MS parameters and the resultant gas-chromatogram is presented in Fig. 8.

The compositions of the phytochemical compounds present in methanolic extracts of DME identified by GC-MS analysis with their retention time (RT), molecular formula, molecular weight and area (%) are presented in (Table 2). An additional file shows the biological and pharmacological activities of identified components in more detail (see Additional file 2).

**Table 2:** List of compounds identified in GC-MS analysis

Sl No.	Name of compounds	Chemical formula	Molecular Weight	Retention time	Area%	Similarity index
1.	Silane, ethenylethoxydimethyl-	C_6_H_14_OSi_2_	130	8.227	1.63	78
2.	4H-Pyran-4-one, 2,3-dihydro-3,5-dihydroxy-6-methyl-	C_6_H_8_O_4_	144	9.357	1.11	94
3.	Catechol	C_6_H_6_O_2_	110	10.333	5.07	95
4.	5-Hydroxymethylfurfural	C_6_H_6_O_3_	126	10.743	1.32	90
5.	Benzeneacetic acid	C_8_H_8_O_2_	136	11.050	0.27	85
6.	Beta.-d-Ribopyranoside, methyl, 3-acetate	C_8_H_14_O_6_	206	14.807	0.40	78
7.	Dodecanoic acid	C_12_H_24_O_2_	200	15.083	0.50	95
8.	Benzenepropanoic acid, 4-hydroxy-, methyl ester	C_10_H_12_O_3_	180	15.377	0.34	85
9.	1,3,4,5-tetrahydroxycyclohexanecarboxylic acid	C_7_H_12_O_6_	192	16.780	4.44	85
10.	Tetradecanoic acid	C_14_H_28_O_2_	228	17.367	0.88	95
11.	1,2-Benzenedicarboxylic acid, bis(2-methylpropyl) ester	C_16_H_22_O_4_	278	18.400	1.14	97
12.	n-Pentadecanol	C_15_H_32_O	228	18.590	0.29	97
13.	Hexadecanoic acid, methyl ester	C_17_H_34_O_2_	270	19.017	0.91	96
14.	2-hydroxycyclopentadecanone	C_15_H_28_O_2_	240	19.233	0.47	85
15.	Dibutyl phthalate	C_16_H_22_O_4_	278	19.363	0.17	95
16.	n-Hexadecanoic acid	C_16_H_32_O_2_	256	19.507	7.30	96
17.	Alpha.-D-Glucopyranoside, methyl	C_7_H_14_O_6_	194	20.203	0.79	84
18.	n-Nonadecanol-1	C_19_H_40_O	284	20.593	1.11	97
19.	9,12-Octadecadienoic acid (Z,Z)-, methyl ester	C_19_H_34_O_2_	294	20.657	0.58	95
20.	6-Octadecenoic acid, methyl ester, (Z)-	C_19_H_36_O_2_	296	20.717	0.52	93
21.	cis-9-Hexadecenal	C_16_H_30_O	238	21.167	6.04	91
22.	Octadecanoic acid	C_18_H_36_O_2_	284	21.347	0.80	93
23.	4,8,12,16-Tetramethylheptadecan-4-olide	C_21_H_40_O_2_	324	22.950	0.23	96
24	1-Heptacosanol	C_27_H_56_O	396	24.093	0.47	95
25.	Hexadecanoic acid, 2-hydroxy-1-(hydroxymethyl)ethyl ester	C_19_H_38_O_4_	330	24.273	2.32	92
26.	Bis(2-ethylhexyl) phthalate	C_24_H_38_O_4_	390	24.407	0.30	96
27.	9,12-Octadecadienoic acid (Z,Z)-, 2-hydroxy-1-hydroxymethyl)ethyl ester	C_21_H_38_O_4_	354	25.690	3.98	91
28.	Squalene	C_30_H_50_	410	26.753	0.76	97
29.	Gamma-Tocopherol	C_28_H_48_O_2_	416	29.767	0.40	94
30.	Vitamin E	C_29_H_50_O_2_	430	31.090	2.01	95
31.	A’-neogammacer-22(29)-ene	C_30_H_50_	410	31.797	0.59	83
32.	1-phenanthrenecarboxylic acid, 7-ethyl-1,2,3,4,4a,4b,5,6,7,9,10,10a-dodecahydro-1,4a,7-trimethyl-, methyl Ester,	C_21_H_34_O_2_	318	32.357	6.07	65
33.	Ergost-5-en-3-ol, (3.beta.,24r)	C_28_H_48_O	400	33.110	1.32	93
34.	Cholest-5-en-3-ol, 4,4-dimethyl-, (3.beta.)	C_29_H_50_O	414	33.703	4.17	82
35.	Ergosta-8,24(28)-dien-3-ol, 4,14-dimethyl-, (3.beta.,4.alpha.,5.alpha.)-	C_30_H_50_O	426	34.603	3.07	86
36.	Stigmast-5-en-3-ol, (3.beta.)	C_29_H_50_O	414	35.180	4.98	81
37.	Cholest-5-en-3-ol, 4,4-dimethyl-, (3.beta.)-	C_29_H_50_O	414	36.577	0.44	78
38.	9,19-Cyclolanost-24-en-3-ol, (3.beta.)-	C_30_H_50_O	426	37.313	1.34	91
39.	9,19-Cyclolanost-25-en-3-ol, 24-methyl-, (3.beta.,24S)-	C_31_H_52_O	440	38.407	0.65	78
40.	(1,5-dimethyl-hexyl)-3a,10,10,12b-tetramethyl-1,2,3,3a,4,6,8,9,10,10a,11,12,12a,12b-tetradecahydro-benzo[4,5]cyclohepta[1,2-e]inde	C_30_H_5_O	410	38.833	1.34	82
41.	Cyclopropa[5,6]-33-norgorgostan-3-ol, 3′,6-dihydro-, (3.beta.,5.beta.,6.alpha.,22.xi.,23.xi.)-	C_30_H_50_O	426	39.143	3.41	79
42.	9,19-Cyclolanostan-3-ol, 24-methylene-, (3.beta.)-	C_31_H_52_O	440	40.447	0.89	83
43.	5-(7a-Isopropenyl-4,5-dimethyl-octahydroinden-4-yl)-3-methyl-pent-2-en-1-ol	C_20_H_34_O	290	41.147	1.29	80
44.	9,19-cyclolanostan-3-ol, 24-methylene-, (3.beta.)-	C_31_H_52_O	440	41.843	0.78	90
44.	9,19-Cyclolanostan-3-ol, 24-methylene-, (3.beta.)-	C_31_H_52_O	440	42.387	1.49	91
45.	Lanost-8-ene-3,22,23-triol, 24-methylene-, 22-acetate, (3.beta.,22r,23 s)-	C_33_H_54_O_4_	514	44.267	0.98	56
46.	9,19-Cyclolanostan-3-ol, 24-methylene-, (3.beta.)-	C_31_H_52_O	440	44.943	0.80	83
47.	9,19-cycloergost-24(28)-en-3-ol, 4,14-dimethyl-, (3.beta.,4.alpha.,5.alpha.)	C_30_H_50_O	426	48.603	0.78	79

#### Molecular docking study

**Fig. 9:**
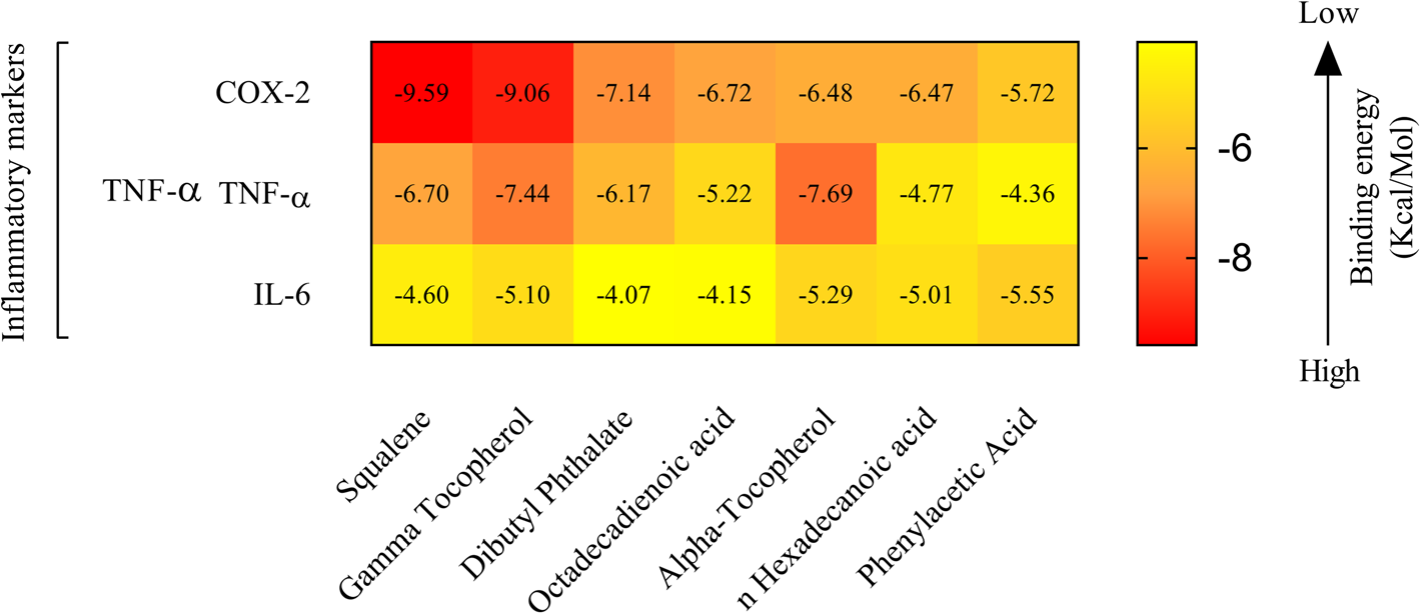
Heat Map representing the binding energies generated by the docking analysis of the active phyto-components against the pro-inflammatory markers

Amongst the various bio-active components identified through GC-MS analysis, 7 components were chosen for further in silico docking study based on their previously reported anti-inflammatory activities, following an extensive literature survey (see Additional file 2). The docking-based study reflected the binding affinity of the 7 chosen ligands to the active site region of COX-2, TNF-α and IL-6 receptor as seen in heat map representation (Fig. 9). An additional file shows the binding energy and binding interactions in more detail (see Additional file 3).

**Fig. 10:**
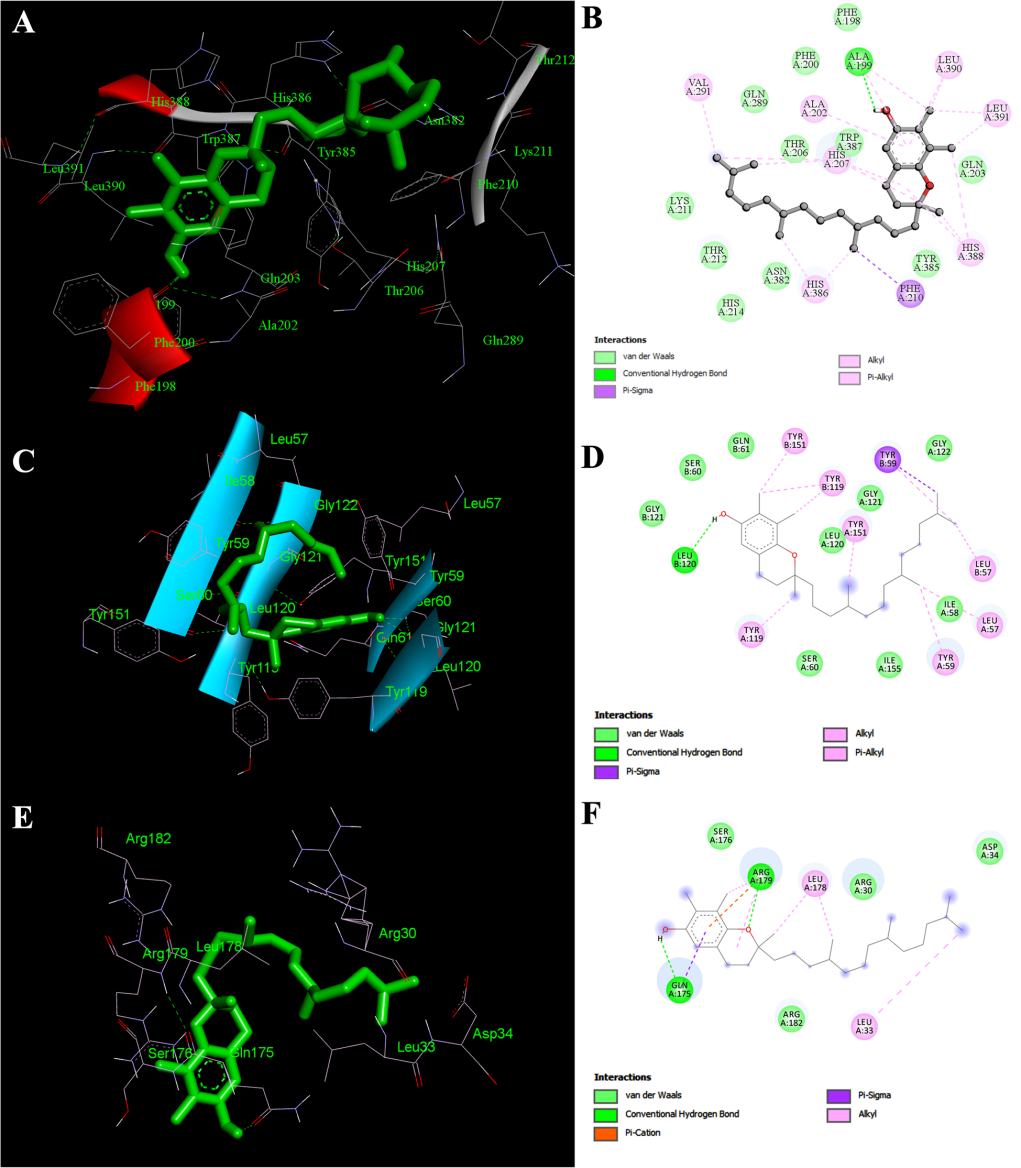
The 3D diagram showing the interactions between the target protein residues and the inhibitor, Gamma Tocopherol (green). In 3D interaction, gamma tocopherol complex with the active site regions of COX-2 (**a**), with the active site regions of TNF-α (**c**) and the active site region of IL-6 (**e**). In 2D interactions, green dotted line represents the hydrogen bond formation between the ligand and the COX-2 enzyme (**b**), TNF-α protein (**d**) and IL-6 protein (**f**)

In COX-2 docking studies, Squalene showed the highest binding affinity (− 9.59 Kcal/Mol) with a 0.094 μM inhibition constant whereas, Phenylacetic Acid showed the least binding affinity (− 5.72 Kcal/Mol) with a 64.12 μM inhibition constant. Squalene also formed several hydrophobic interactions (at ALA 199, ALA 202, GLN 203, THR 206, HIS 207, PHE 210, LYS 211, THR 212, GLN 289, VAL 291, ASN 382, HIS 386, TRP 387, HIS 388, LEU 390 and LEU 391 residues) with the target protein by covering the active site at tyrosine 385 residue). Squalene also showed a good binding energy with the target protein IL-6 (− 4.60 Kcal/Mol) and TNF-α (− 6.70 Kcal/Mol) with several hydrophobic interactions (see Additional file 3). Lack of hydroxyl groups in squalene, the hydrophobic interactions plays an important role in COX-2 and in both IL-6 and TNF-α inhibition activity. Gamma tocopherol showed the second-highest binding affinity (9.06 Kcal/Mol) with a 0.228 μM inhibition constant and formed one hydrogen (H) bond with the target protein COX-2 at alanine 199 residue (Fig. 10a and b). It also formed a number of hydrophobic contracts (at ALA 202, HIS 207, PHE 210, VAL 291, HIS 386, HIS 388, LEU 390, and LEU 391 residues) that contribute to its binding energy thus reflecting its COX-2 inhibition activity. It also formed one H bond (at Leu 120 residue) with the target protein TNF-α (Fig. 10c and d) and several hydrophobic interactions (at LEU 57, ILE 58, TYR 59, SER 60, GLN 61, TYR 119, GLY 121, GLY 122, TYR 151, ILE 155) reflected the second highest inhibitory binding energy (− 7.44 Kcal/Mol, inhibition constant 3.50 μM). It also showed a good binding energy (− 5.10 Kcal/Mol) and two H bond (at GLN 175, ARG 179 residues) formation with the target protein IL-6 (Fig. 10 e and f) with a number of hydrophobic interactions (at ARG 30, LEU 33, ASP 34, SER 176, LEU 178, ARG 182 residues) that contributed the inhibition activity (183.63 μM).

Similarly, Dibutyl phthalate and n-Hexadecanoic acid showed good binding energy (− 7.14 Kcal/Mol and − 6.47 Kcal/Mol respectively) with two H bonds formations at target protein COX-2 (at HIS 207, HIS 388 residues and ARG 120 and TYR 355 respectively). Dibutyl phthalate and n-Hexadecanoic acid also formed two H bonds (both at ARG 179 ARG 182 residues) at target protein IL-6 with a − 4.07 Kcal/Mol and − 5.01 Kcal/Mol binding energy respectively. Each phyto-component formed one H bond (both at TYR 151 residues) at target protein TNF-α with a good binding energy (− 6.17 Kcal/Mol and − 4.77Kcal/Mol respectively) with several hydrophobic interactions (see Additional file 3). Alpha-Tocopherol showed the highest binding affinity with TNF-α (− 7.69 Kcal/Mol) and second highest binding affinity with IL-6 (− 5.55 Kcal/Mol) by forming 2 H bonds (at GLN 175, and ARG 179 residues). It also showed a good binding energy (− 6.48 Kcal/Mol) with an array of hydrophobic bond formation (at HIS 90, ARG 120, VAL 349, LEU 352, SER 353, LEU 359, PHE 381, LEU 384, TYR 385, TRP 387, ARG 513, PHE 518, MET 522, VAL 523, GLY 526, ALA 527, SER 530, LEU 531) with the target protein COX-2. Another phyto-component, Phenylacetic acid showed − 4.36 Kcal/Mol binding energy with the most H bond formation (at LYS 98, PRO 117, TYR 119 residues) at the target protein TNF-α and showed a good binding energy (− 5.72 Kcal/Mol) with the target protein COX-2. Phenylacetic acid also showed the highest binding energy (− 5.55 Kcal/Mol) with the target protein IL-6 and formed two H bonds (at ARG 179 and ARG 182 residues) and several hydrophobic interactions. An additional file shows all structures in 3D (see Additional file 4).

## Discussion

The anti-inflammatory role of the *Drynaria quercifolia* methanolic extract (DME) has been assessed in detail in the different in vitro, in vivo and in silico systems and the results have been corroborated. Protein denaturation and membrane stabilization tests are efficient methods to screen the potency of drug molecules as well as herbal extracts as potent anti-inflammatory agents. Most biological proteins lose their three-dimensional integrity as well as their biological function following the heat-exposure and play a vital role to induce hypersensitivity and chronic inflammatory arthritis [34, 50, 51]. Other key mediators involved in the systemic inflammatory process are the lysosomal enzymes. A stabilized membrane prevents inflammatory progression by lowering the oxidative damage [34]. Injury to the lysosomal membrane renders the cell more vulnerable to secondary damage by releasing the lysosomal contents [51]. It is well documented that the human red blood cell membrane shows a structural similarity to the lysosomal membrane [50, 51]. Hence, it is postulated that any expected drug offering significant protection over heat-induced protein alteration and the haemolysis process of erythrocytes, will be delivered the anti-inflammatory property in the inflamed body of the host. In our experiments, DME showed a dose-dependent significant inhibition of denatured protein and a protective effect on membrane stabilization on both the hypotonicity and heat induced damage (Table 1). DME could possibly restore protein integrity by stabilizing the changes in electrostatic, hydrogen, hydrophobic and disulfide-bonding and by preventing the rupture of a membrane protein by expanding surface-volume ratio and also by preventing the release of inflammatory mediators [32, 51].

Assessment and evaluation of the toxic characteristic of medicinal plant-product is an initial step in screening the pharmacological activity of natural products. In the acute oral toxicity test, DME also showed no toxic effects in experimental animal groups up to the used highest dose group of 2000 mg/kg body weight following a 14 day-long observation. All these aforementioned observations served as a basis for choosing the dose selection for in vivo studies.

The FCA-induced arthritis model in rats is one of the most used models to study the anti-rheumatic properties of various compounds as it provides similar clinical and immunological characteristics to human RA [36, 52, 53]. The measurement of rat paw circumference was used to assess the degree of inflammation and to depict the possible therapeutic effects of DME on experimental rats. The measurement of rat paw circumference clearly showed the reduction in the paw-swelling in experimental groups at the end of the experimental schedule (Fig. 2). In arthritic conditions, RBC counts decreases because bone marrow is depressed and fails to respond to anemic condition**.** Our results also show a decreased level of RBC count in arthritic control groups. This may be due to the abnormal storage of iron or the inability of bone marrow to produce cells [53]. However, the RBC count increases significantly towards their normal ranges in both the low and high dose groups when treated with DME (Fig. 3b). The hemoglobin count also decreases in arthritic condition as low RBC level results in low hemoglobin levels. The significant increases of WBC count in arthritic condition account for the boosting of the immune system with the invading antigens [54]. A significant decrement of platelet counts was also observed in the experimental groups when compared to arthritic control groups (Fig. 3d). Thus, treatments with DME normalized the haematological parameters in the experimental groups which reflect its immunomodulatory effects.

In the present study, we have observed a decrease in serum total protein level (Fig. 4a) and albumin level (Fig. 4b) in the arthritic control rats. This due to the changes in plasma protein concentration and involvement of several inflammatory mediators like prostaglandins, bradykinin that increases the vascular permeability leads to the reduction of total protein and albumin levels [54]**.** Treatment of DME significantly increases the total protein level in both experimental groups when compared to the arthritic control group (Fig. 4a). Furthermore, the high dose group rats showed a significant increase in the albumin level when compared with the rats of the arthritic control group suggesting the role of DME in the restoration of serum albumin level (Fig. 4b), which may indicate the suppressive activity of DME on the inflammatory mediators. However, in our study, no significant alteration was observed in the serum creatinine level in all the experimental groups (Fig. 4c), which indicates normal kidney function. Ceruloplasmin is another major acute-phase serum protein that increases during RA [36]. This copper-containing protein is mainly produced in the liver and released in the blood circulation upon tissue injury [54]. We have observed an elevated level of this protein in the arthritic control group which indicates the tissue injury during the chronic arthritis progression (Fig. 4d). However, a significant decrease was observed in both the low and high dose groups after the DME treatment (Fig. 4d), which shows its effects on tissue repair.

In clinical practice, RA diagnosis and follow-up are mainly based on conventional radiographs, which are useful diagnostic measures in RA severity. The most common earliest sign of RA is the soft tissue swelling, whereas in more chronic stages, is the narrowing of joint spaces and erosions of bones occurs [55]. In our study, we have observed a soft tissue swelling along with irregular joint space in arthritic control groups, that may indicate the severity of the disease progression (Fig. 5d and h). However, DME treatment for 28 days has shown the prevention of bone destruction in both the low (Fig. 5b and f) and high dose groups (Fig. 5c and g), when compared to arthritic control groups. These radiographic changes are further supported by histopathological analysis of ankle-joints of the experimental rats. In the arthritic control groups, the arthritic rat joint showed extensive infiltration of inflammatory cells in the articular cartilage regions (Fig. 6d). However, treatment with DME for 28 days showed less cellular infiltrations in both the low and high dose groups, when compared to the arthritic control group animals.

GC-MS technique is routinely employed to identify the array of volatile bioactive compounds present in a plant extract [56, 57]. The GC-MS analysis of the DME detected several promising compounds (Fig. 8). The detailed analysis of chromatogram has revealed the presence of 47 potent bio-active compounds in the methanolic rhizome extract of *Drynaria quercifolia* (Table 2). Compounds like squalene can attenuate the overexpressed COX-2 activity in Lipopolysaccharides (LPS) treated human monocytes and neutrophils and also down-regulate Matrix metalloproteinase (MMP)-1 and MMP-9 gene expression in LPS treated human monocytes, and of MMP-1 and MMP-3 gene expression in LPS-treated human neutrophils [58] and it also has a potent antioxidant and anticancer activity [59]. Similarly, it has been previously reported that compounds like n-Hexadecanoic acid and Gamma-tocopherol also possess a potent anti-inflammatory activity [60–62].

Our in vitro and in vivo analysis of anti-inflammatory and anti-rheumatoid activity of DME showed promising results. To further substantiate the results in silico, molecular docking technique was utilized to verify the ability of phyto-constituents to inhibit principal inflammatory mediators such as COX-2, TNF-α and IL-6. COX-2 is the main enzyme that mediates the bioconversion of arachidonic acid to inflammatory prostaglandins [44]. The arachidonate binding site is created by a long hydrophobic channel, from Arg 120 to near Tyr 385 and Glu 524, and this binding channel provides the binding pockets of traditional NSAIDs for selective inhibition [63].

Molecular docking is one of the most powerful techniques to discover novel ligands for proteins of known structure and thus plays a key role in structure-based drug design. It is based on the “lock-key” principle, which looks into the ligand-receptor interaction through the formation of electrostatic interaction, hydrogen bonding, hydrophobic interaction, van der Waals interaction, etc. In this study, seven phytochemical compounds identified from DME were docked with COX-2, TNF-α, IL-6 proteins to see whether these compounds can bind to the active sites of the enzymes and inhibit their activities which may indicate the anti-inflammatory activity of DME.

In our docking assays, the binding energy and inhibition constant obtained from the calculated results by Autodock 4.2 using a Lamarckian genetic algorithm [49], was the reflection of the binding affinity of the bioactive ligands with the target proteins. The smaller the inhibition constant, the greater the binding affinity and the smaller amount of medication needed to inhibit the activity of that enzyme. Compounds like Squalene, Phenylacetic acid, α-Tocopherol, n-Hexadecanoic acid, γ-Tocopherol, Dibutyl phthalate showed efficient binding affinity with the target inflammatory markers which may subsequently down-regulate inflammation. COX-2 inhibition leads to a decrease in the production of prostaglandin E2 which is a limiting factor in inflammation [44, 64]. Docking of the phyto-compounds with the target proteins are explored in detail to reveal the number of hydrogen bonds in the interaction and the interacting amino acids. Recent studies on plant extract and herbal products have revealed that the plant-based herbal remedies can directly act on the transcription of specific genes responsible for the production of cytokines, cyclooxygenases and other inflammatory biomarkers [10, 65, 66]. In a recent study, the present author group has demonstrated that *Aloe vera* gel in its crude form, may downregulate TNF-α and COX-2 expressions in Wistar rat inflammatory arthritis models [10]. The in silico study indicates that the target inflammatory markers have been inhibited significantly by the phyto-compounds of the *Drynaria quercifolia* as indentified in GC-MS study. The authors assume that similar inhibition of COX-2 may be possible in vivo at protein and/or gene expression levels.

In inflammatory arthritis, the infiltrating immune cells contribute to the degree of inflammation [5, 8]. The initial infiltrating cells stimulate both Th1 and Th2 cell types; Th1 cells provide delayed type of hypersensitivity and Th2 involves B-cells in the inflammatory process [67]. A vast array of cytokines including TNF-α, interleukins and other inflammation mediators like COX-2 are secreted from the infiltrating immune cells and synoviocytes during the disease course [8]. The blocking of these key molecules may provide an excellent interruption in the progress of inflammation. As seen in our studies, significant synergistic interactions between *Drynaria* phyto-compounds with cytokines like TNF-α, IL-6 and with COX-2-like immune-modulators may play significant roles in down-regulating inflammation. Some phyto-components have been shown to interact with cytokines and some others with COX-2 in controlling progression of the disease. In this regard, the known phyto-compounds of *Withania somnifera* has shown interaction with different cytokines in in silico docking and in vivo animal models and downregulated the expressions of iNOS, TNF-α, NF-κB etc. [68, 69]. Compounds from *Canabis sative* and *Prunella vulgaris* also showed interactions with different cytokines in silico [68]. *Moringa rivae* leaf extract is known to down-regulate the TNF- α and COX-2 expressions in the animal model systems [65]; Fenugreek (*Trigonella foenum graecum*), extracted in ethanol, ameliorates arthritis in experimental animals and decreased TNF-α expression [70]. Poly-herbal formulations like Kashayams have been shown to down-regulate elevated levels of Cox-2, TNF-α, iNOS mRNAs in vivo when treated to arthritic rat models [71].

Therefore, it can be inferred that the anti-inflammatory and anti-rheumatoid effect of methanolic rhizome extract of *Drynaria quercifolia* may be due to the presence of potent bio-active components which may synergistically show the inhibitory effects on inflammatory markers (COX-2, TNF-α and IL-6) leading to inhibition of inflammation.

## Concluding remarks

Our study experimentally presents the anti-inflammatory and anti-arthritic activities of *Drynaria quercifolia* rhizome methanolic extract in both in vitro and in vivo scenarios. Both the membrane stabilization and protein denaturation test confirmed the anti-inflammatory activity of extracts. Our results further validated the ameliorative property of *D. quercifolia* in FCA-induced chronic arthritis model. Treatment with the plant extracts improved the inflammatory paw edema, hematological and biochemical parameters without exhibiting any signs of hepatotoxicity and nephrotoxicity. Moreover, GC–MS analysis indicated that the bio-active phyto-components of *D. quercifolia* possess potent anti-inflammatory activity against molecular targets. The results of the docking study provide validation of anti-inflammatory activity. However, an extensive screening and identification of the major anti-inflammatory phyto-components is also required involving their isolation, purification, bio-availability assays. Further studies regarding the role of the plant-extract and its phyto-compounds on the gene and protein-level expression of a broad spectrum of inflammatory biomarkers would provide a clearer view on the anti-inflammatory aspects of the plant. Furthermore, the toxicological properties of the plant extract and its components also need a detailed exploration. The study outcome denotes that the *D. quercifolia* rhizome methanolic extract can provide a new opportunity for finding a complementary and alternative remedy in inflammatory and arthritic disease conditions.

## Supplementary Information

**Additional file 1.** Flow sheet of methodology.

**Additional file 2.** List of compounds identified in GC-MS analysis with their chemical class and biological properties.

**Additional file 3: Table S1.** Molecular docking scores of bioactive compounds identified in Drynaria quercifolia methanolic extracts with COX-2, IL-6, and TNF-α.

**Additional file 4 Figure S1.** Represents the interaction between the COX-2 and the inhibitor, Squalene. **Figure 2.** Represents the interaction between the COX-2 and the inhibitor, Dibutyl phthalate. **Figure 3.** Represents the interaction between the COX-2 and the inhibitor, 9,12-Octadecadienoic acid (Z,Z)-, methyl ester_Methyl Linoleate. **Figure 4.** Represents the interaction between the COX-2 and the inhibitor, Vitamin E, Alpha –Tocopherol. **Figure 5.** Represents the interaction between the COX-2 and the inhibitor, n Hexadecanoic acid. **Figure 6.** represents the interaction between the COX-2 and the inhibitor, Phenylacetic Acid. **Figure 7.** represents the interaction between the TNF-α and the inhibitor, Squalene. **Figure 8.** Represents the interaction between the TNF-α and the inhibitor, Dibutyl phthalate. **Figure 9.** Represents the interaction between the TNF-α and the inhibitor, 9,12-Octadecadienoic acid (Z,Z)-, methyl ester_Methyl Linoleate. **Figure 10.** Represents the interaction between the TNF-α and the inhibitor, Vitamin E, Alpha –Tocopherol. **Figure 11.** Represents the interaction between the TNF-α and the inhibitor, n Hexadecanoic acid. **Figure 12.** Represents the interaction between the TNF-α and the inhibitor, n Phenylacetic Acid. **Figure 13.** Represents the interaction between the IL-6 and the inhibitor, Squalene. **Figure 14.** Represents the interaction between the IL-6 and the inhibitor, Dibutyl Phthalate. **Figure 15.** Represents the interaction between the IL-6 and the inhibitor, 9,12-Octadecadienoic acid (Z,Z)-, methyl ester_Methyl Linoleate. **Figure 16.** Represents the interaction between the IL-6 and the inhibitor, Vitamin E, Alpha –Tocopherol. **Figure 17.** Represents the interaction between the IL-6 and the inhibitor, n Hexadecanoic acid. **Figure 18.** Represents the interaction between the IL-6 and the inhibitor, Phenylacetic Acid.

## Data Availability

All the materials will be available for research purposes if requested to the corresponding author.

## Abbreviations

ANOVA: Analysis of variance
b.w.: Body weight
CPCSEA: Committee for the Purpose of Control and Supervision of Experiments on Animals
COX-2: Cyclooxygenase-2
DMARDs: Disease-Modifying Anti-Rheumatic Drugs
DME: *Drynaria quercifolia* rhizome Methanolic Extract
DMSO: Dimethyl sulfoxide
EDTA: Ethylene-diamine-tetraacetic acid
FCA: Freund’s Complete Adjuvant
GA: Genetic algorithm
GC-MS: Gas Chromatography-Mass Spectrometry
HCl: Hydrogen chloride
IAEC: Institutional Animal Ethical Committee
i.p.: Intraperitoneal
LPS: Lipopolysaccharides
MMP: Matrix metalloproteinase
NCBI: National Center for Biotechnology Information
NSAIDs: Non-Steroidal Anti-Rheumatic Drugs
OECD: Organization for Economic Cooperation and Development
PBS: Phosphate-buffered saline
p.o.: Per os
RA: Rheumatoid Arthritis
RBC: Red Blood Corpuscles
S.D.: Standard deviation
S.E.M: Standard Error Mean
TIC: Total ion count
WBC: White Blood Corpuscles
