# Supplementary material for: Validating potent anti-inflammatory and anti-rheumatoid properties of Drynaria quercifolia rhizome methanolic extract through in vitro, in vivo, in silico and GC-MS-based profiling
Source: BMC Complement Med Ther. 2021 Mar 12;21:89. doi: 10.1186/s12906-021-03265-7 (PMC7953762; doi:10.1186/s12906-021-03265-7)

TITLE

Validating potent anti-inflammatory and anti-rheumatoid properties of *Drynaria quercifolia* rhizome methanolic extract through *in vitro, in vivo, in silico and* GC-MS-based profiling.

Authors:

Debabrata Modak[1], Subhashis Paul[1], Sourav Sarkar[1], Subarna Thakur[2] and Soumen Bhattacharjee[1*].

[1]Cell and Molecular Biology Laboratory, Department of Zoology, University of North Bengal, Darjeeling 734013, West Bengal, India.

[2]Department of Bioinformatics, University of North Bengal, Darjeeling 734013, West Bengal, India.

Title: Additional file 1

Description: Flow sheet of methodology.

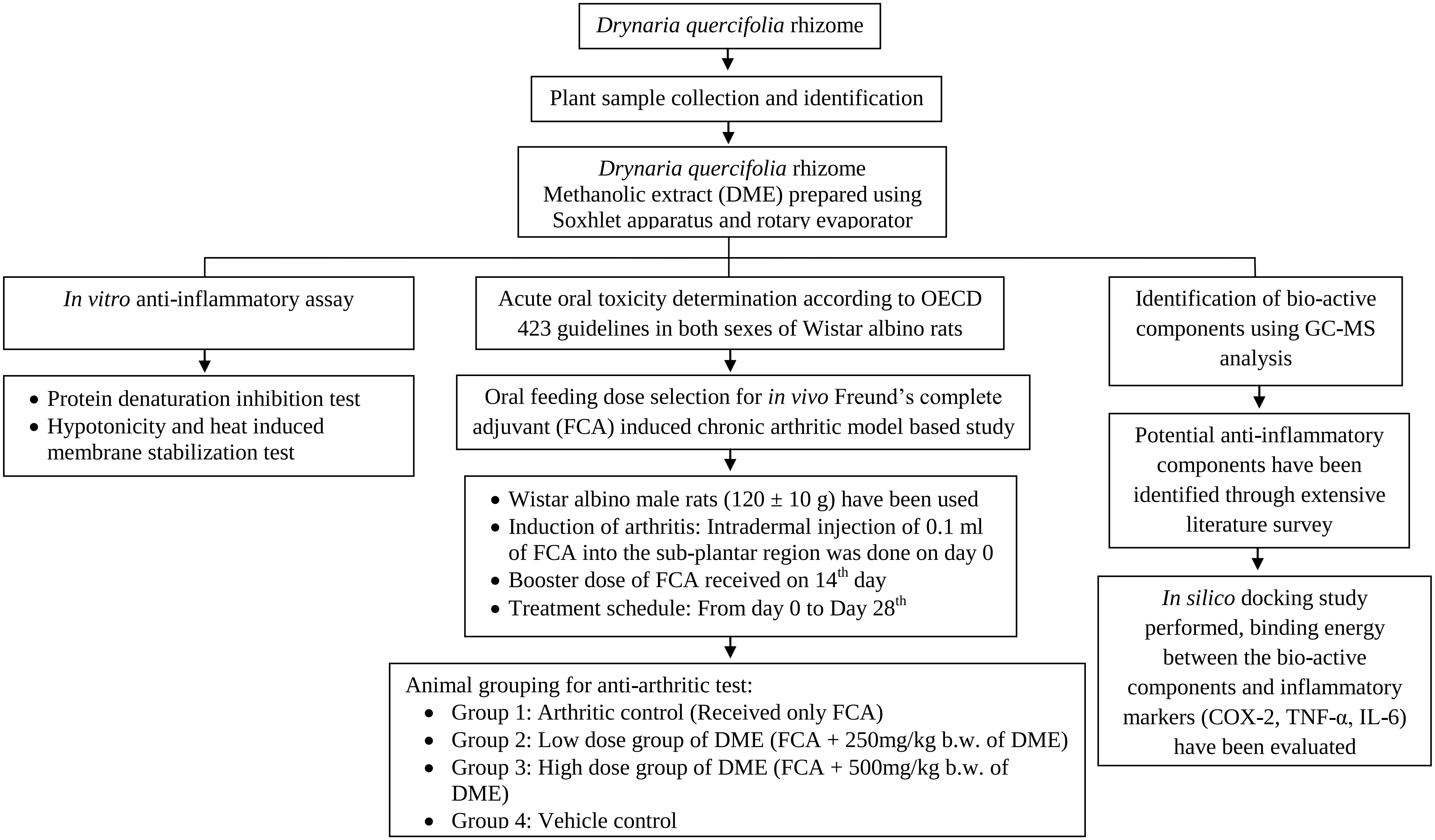

Supplement: Supplementary file 1 — Additional file 1. Flow sheet of methodology. [file 12906_2021_3265_MOESM1_ESM.pdf]
