# Supplementary material for: Validating potent anti-inflammatory and anti-rheumatoid properties of Drynaria quercifolia rhizome methanolic extract through in vitro, in vivo, in silico and GC-MS-based profiling
Source: BMC Complement Med Ther. 2021 Mar 12;21:89. doi: 10.1186/s12906-021-03265-7 (PMC7953762; doi:10.1186/s12906-021-03265-7)
Supplement: Supplementary file 2 — Additional file 2. List of compounds identified in GC-MS analysis with their chemical class and biological properties. [file 12906_2021_3265_MOESM2_ESM.pdf]

TITLE

Validating potent anti-inflammatory and anti-rheumatoid properties of *Drynaria quercifolia* rhizome methanolic extract through *in vitro, in vivo, in silico and* GC-MS-based profiling.

Authors:

Debabrata Modak[1], Subhashis Paul[1], Sourav Sarkar[1], Subarna Thakur[2] and Soumen Bhattacharjee[1*].

[1]Cell and Molecular Biology Laboratory, Department of Zoology, University of North Bengal, Darjeeling 734013, West Bengal, India.

[2]Department of Bioinformatics, University of North Bengal, Darjeeling 734013, West Bengal, India.

Title: Additional file 2

Description: List of compounds identified in GC-MS analysis with their chemical class and biological properties.

Sl No.	Compound Name with formula	Chemical class	Molecular Weight (kDa); Retention Time (second)	Area%	SI	Biological Properties with Reference
1.	Silane, ethenylethoxydimethyl- [$C_6H_{14}OSi_2$]	Group 14 hydride	130; 8.227	1.63	78	Not reported
2.	4H-Pyran-4-one, 2,3-dihydro-3,5-dihydroxy-6-methyl- [$C_6H_8O_4$]	Pyranone	144; 9.357	1.11	94	Anti-proliferative and pro-apoptotic [1],Antioxidant [2], Modulate autonomic nerve activity in Rat model [3]
3.	Catechol [$C_6H_6O_2$]	Benzenediols	110; 10.333	5.07	95	Antimicrobial [4]
4.	5-Hydroxymethylfurfural [$C_6H_6O_3$]	Furans	126; 10.743	1.32	90	Antioxidant and Antiproliferative [5], Inhibitory effect on the sickling of red blood cells [6], Anti-allergic (Type I allergic Diseases [7],Anti-inflammatory [8]
5.	Benzeneacetic acid [$C_8H_8O_2$]	Monocarboxylic acid containing a phenyl group	136; 11.050	0.27	85	Antimicrobial [9], Anti-inflammatory [10], Derivatives of phenylacetic acids [11]
6.	Beta.-d-Ribopyranoside, methyl, 3-acetate [$C_8H_{14}O_6$]	D-ribopyranose	206; 14.807	0.40	78	Not reported
7.	Dodecanoic acid [$C_{12}H_{24}O$]	Saturated fatty acid	200; 15.083	0.50	95	Not reported
8.	Benzenepropanoic acid, 4-hydroxy-, methyl ester [$C_{10}H_{12}O_3$]	Phenyl propanoids	180; 15.377	0.34	85	Not reported

9.	1,3,4,5-tetrahydroxycyclohexane carboxylic acid [$C_7H_{12}O_6$]	Cyclitol and cyclohexanecarboxylic acid	192; 16.780	4.44	85	Not reported
10.	Tetradecanoic acid [$C_{14}H_{28}O_2$]	Saturated fatty acid	228; 17.367	0.88	95	Larvicidal and repellent [12]
11.	1,2-Benzenedicarboxylic acid, bis(2-methylpropyl) ester [$C_{16}H_{22}O_4$]	Benzoic acid esters	278; 18.400	1.14	97	Not reported
12.	n-Pentadecanol [$C_{15}H_{32}O$]	Saturated long-chain fatty alcohol	228; 18.590	0.29	97	Antimicrobial [13]
13.	Hexadecanoic acid, methyl ester [$C_{17}H_{34}O_2$]	Saturated fatty acid	270; 19.017	0.91	96	Anti-inflammatory, Anti microbial, Antioxidant [14,15]
14.	2-hydroxycyclopentadecanone [$C_{15}H_{28}O_2$]	Cyclopentadecanone	240; 19.233	0.47	85	Not reported
15.	Dibutyl phthalate [$C_{16}H_{22}O_4$]	Phthalates	278; 19.363	0.17	95	Antimicrobial, Anti-inflammatory [16,17]
16.	n-Hexadecanoic acid [$C_{16}H_{32}O_2$]	Saturated fatty acid	256; 19.507	7.30	96	Anti-bacterial, Anti-fungal [18]
17.	Alpha.-D-Glucopyranoside, methyl [$C_7H_{14}O_6$]	Monosaccharide	194; 20.203	0.79	84	Growth inhibitory effect on mammalian cells and parasites [19]
18.	n-Nonadecanol-1 [$C_{19}H_{40}O$]	Long-chain fatty alcohols	284; 20.593	1.11	97	Antimicrobial, Cytotoxic activity [20]
19.	9,12-Octadecadienoic acid (Z,Z)-, methyl ester [$C_{19}H_{34}O_2$]	Polyunsaturated Omega-6 trans fatty acid (TFA)	294; 20.657	0.58	95	Anti-inflammatory, Anti-cancer [21,22]
20.	6-Octadecenoic acid, methyl ester, (Z)- [$C_{19}H_{36}O_2$]	Monounsaturated omega-12 fatty acid	296; 20.717	0.52	93	Not reported
21.	cis-9-Hexadecenal [$C_{16}H_{30}O$]	Monounsaturated omega-7 fatty acid	238; 21.167	6.04	91	Antimicrobial [23]
22.	Octadecanoic acid [$C_{18}H_{36}O_2$]	Saturated fatty acid	284; 21.347	0.80	93	Antimicrobial [24]
23.	4,8,12,16-Tetramethylheptadecan-4-olide [$C_{21}H_{40}O_2$]	Long-chain methyl-branched fatty acid	324; 22.950	0.23	96	Not reported
24	1-Heptacosanol [$C_{27}H_{56}O$]	Long-chain primary fatty alcohol	396; 24.093	0.47	95	Antimicrobial [20]
25.	Hexadecanoic acid, 2-	2-monoglyceride	330;	2.32	92	Not reported

	hydroxy-1-(hydroxymethyl)ethyl ester [$C_{19}H_{38}O_4$]		24.273			
26.	Bis(2-ethylhexyl) phthalate [$C_{24}H_{38}O_4$]	Diester of phthalic acid and the branched-chain **2-**ethylhexanol	390; 24.407	0.30	96	Antimicrobial, Cytotoxic activity [25]
27.	9,12-Octadecadienoic acid (Z,Z)-, 2-hydroxy-1-hydroxymethyl)ethyl ester [$C_{21}H_{38}O_4$]	2-monoglyceride	354; 25.690	3.98	91	Not reported
28.	Squalene [$C_{30}H_{50}$]	Triterpene	410; 26.753	0.76	97	Anti inflammatory, Anticancer, Antioxidant [26,27,28,29,30]
29.	Gamma.-Tocopherol [$C_{28}H_{48}O_2$]	Tocopherol	416; 29.767	0.40	94	Potent Anti-inflammatory, Anti-cancer [31,32,33,34]
30.	Vitamin E [$C_{29}H_{50}O_2$]	Tocopherol	430; 31.090	2.01	95	Anti-inflammatory, Anti-cancer, Antioxidant, Immunomodulatory, Wound healing [35,36,37,38]
31.	A'-neogammacer-22(29)-ene [$C_{30}H_{50}$]	Triterpene	410; 31.797	0.59	83	Not reported
32.	1-phenanthrenecarboxylic acid, 7-ethyl-1,2,3,4,4a,4b,5,6,7,9,10,10a-dodecahydro-1,4a,7-trimethyl-, methyl Ester, [$C_{21}H_{34}O_2$]	Fatty acid ester	318; 32.357	6.07	65	Not reported
33.	Ergost-5-en-3-ol, (3.beta.,24r) [$C_{28}H_{48}O$]	Steroid	400; 33.110	1.32	93	Not reported
34.	Cholest-5-en-3-ol, 4,4-dimethyl-, (3.beta.) [$C_{29}H_{50}O$]	Steroid	414; 33.703	4.17	82	Not reported
35.	Ergosta-8,24(28)-dien-3-ol, 4,14-dimethyl-, (3.beta.,4.alpha.,5.alpha.)-[$C_{30}H_{50}O$]	3beta-sterol	426; 34.603	3.07	86	Not reported
36.	Stigmast-5-en-3-ol, (3.beta.) [$C_{29}H_{50}O$]	Sterol	414; 35.180	4.98	81	Immunomodulatory,

						Apoptotic, Anti-proliferative [39,40]
37.	Cholest-5-en-3-ol, 4,4-dimethyl-, (3.beta.)-[$C_{29}H_{50}O$]	3beta-sterol	414; 36.577	0.44	78	Antilisterial activity [41]
38.	9,19-Cyclolanost-24-en-3-ol, (3.beta.)-[$C_{30}H_{50}O$]	Triterpenoid	426; 37.313	1.34	91	Not reported
39.	9,19-Cyclolanost-25-en-3-ol, 24-methyl-, (3.beta.,24S)-[$C_{31}H_{52}O$]	Triterpenoid	440; 38.407	0.65	78	Not reported
40.	(1,5-dimethyl-hexyl)-3a,10,10,12b-tetramethyl-1,2,3,3a,4,6,8,9,10,10a,11,12,12a,12b-tetradecahydro-benzo[4,5]cyclohepta[1,2-e]inde [$C_{30}H_{50}O$]	Triterpenoid	410; 38.833	1.34	82	Not reported
41.	Cyclopropa[5,6]-33-norgorgostan-3-ol, 3',6-dihydro-, (3.beta.,5.beta.,6.alpha.,22.xi.,23.xi.)- [$C_{30}H_{50}O$]	Triterpenoid	426; 39.143	3.41	79	Not reported
42.	9,19-Cyclolanostan-3-ol, 24-methylene-, (3.beta.)-[$C_{31}H_{52}O$]	Triterpenoid	440; 40.447	0.89	83	Not reported
43.	5-(7a-Isopropenyl-4,5-dimethyl-octahydroinden-4-yl)-3-methyl-pent-2-en-1-ol [$C_{20}H_{34}O$]	Triterpenoid	290; 41.147	1.29	80	Not reported
44.	9,19-cyclolanostan-3-ol, 24-methylene-, (3.beta.)-[$C_{31}H_{52}O$]	Triterpenoid	440; 41.843	0.78	90	Not reported
44.	9,19-Cyclolanostan-3-ol, 24-methylene-, (3.beta.)-[$C_{31}H_{52}O$]	Triterpenoid	440; 42.387	1.49	91	Not reported
45.	Lanost-8-ene-3,22,23-triol, 24-methylene-, 22-acetate, (3.beta.,22r,23s)-[$C_{33}H_{54}O_4$]	Triterpenoid	514; 44.267	0.98	56	Not reported
46.	9,19-Cyclolanostan-3-ol, 24-methylene-, (3.beta.)-[$C_{31}H_{52}O$]	Triterpenoid	440; 44.943	0.80	83	Not reported
47.	9,19-cycloergost-24(28)-en-3-ol, 4,14-dimethyl-, (3.beta.,4.alpha.,5.alpha.) [$C_{30}H_{50}O$]	Triterpenoid	426; 48.603	0.78	79	Not reported

REFERENCES:

1. Ban JO, Hwang IG, Kim TM, Hwang BY, Lee US, Jeong HS, et al. Anti-proliferate and pro-apoptotic effects of 2, 3-dihydro-3, 5-dihydroxy-6-methyl-4H-pyranone through inactivation of NF-κB in human colon cancer cells. Arch Pharm Res. 2007;30:1455-63.
2. Hwang IG, Kim HY, Woo KS, Lee SH, Lee J, Jeong HS. Isolation and identification of the antioxidant DDMP from heated pear (*Pyrus pyrifolia* Nakai). Prev Nutr Food Sci. 2013;18:76.
3. Beppu Y, Komura H, Izumo T, Horii Y, Shen J, Tanida M, et al. Identificaton of 2, 3-Dihydro-3, 5-dihydroxy-6-methyl-4 H-pyran-4-one Isolated from *Lactobacillus pentosus* Strain S-PT84 Culture Supernatants as a Compound That Stimulates Autonomic Nerve Activities in Rats. J Agric Food Chem. 2012;60:11044-9.
4. Kocaçalışkan I, Talan I, Terzi I. Antimicrobial activity of catechol and pyrogallol as allelochemicals. Z Naturforsch C. 2006;61:639-42.
5. Zhao L, Chen J, Su J, Li L, Hu S, Li B, et al. In vitro antioxidant and antiproliferative activities of 5-hydroxymethylfurfural. J Agric Food Chem. 2013;61:10604-11.
6. Abdulmalik O, Safo MK, Chen Q, Yang J, Brugnara C, Ohene-Frempong K, et al. 5-hydroxymethyl-2-furfural modifies intracellular sickle haemoglobin and inhibits sickling of red blood cells. Br J Haematol. 2005;128:552-61.
7. Yamada P, Nemoto M, Shigemori H, Yokota S, Isoda H. Isolation of 5-(hydroxymethyl) furfural from *Lycium chinense* and its inhibitory effect on the chemical mediator release by basophilic cells. Planta Med. 2011;77:434-40.
8. Kong F, Lee BH, Wei K. 5-Hydroxymethylfurfural Mitigates Lipopolysaccharide-Stimulated Inflammation via Suppression of MAPK, NF-κB and mTOR Activation in RAW 264.7 Cells. Molecules. 2019;24:275.
9. Kim Y, Cho JY, Kuk JH, Moon JH, Cho JI, Kim YC, et al. Identification and antimicrobial activity of phenylacetic acid produced by *Bacillus licheniformis* isolated from fermented soybean, Chungkook-Jang. Curr Microbiol. 2004;48:312-7.
10. Birmingham B, Buvanendran A. Nonsteroidal Anti-inflammatory Drugs, Acetaminophen, and COX-2 Inhibitors. Pract Pain Manag. 2014:553–68. doi:10.1016/b978-0-323-08340-9.00040-2.
11. Tomić M, Micov A, Pecikoza U, Stepanović-Petrović R. Clinical Uses of Nonsteroidal Anti-Inflammatory Drugs (NSAIDs) and Potential Benefits of NSAIDs Modified-Release Preparations. Microsized and Nanosized Carriers for Nonsteroidal Anti-Inflammatory Drugs. 2017:1–29. doi:10.1016/b978-0-12-804017-1.00001-7.
12. Sivakumar R, Jebanesan A, Govindarajan M, Rajasekar P. Larvicidal and repellent activity of tetradecanoic acid against *Aedes aegypti* (Linn.) and Culex *quinquefasciatus* (Say.)(Diptera: Culicidae). Asian Pac J Trop Med. 2011;4:706-10.
13. Togashi N, Shiraishi A, Nishizaka M, Matsuoka K, Endo K, Hamashima H, et al. Antibacterial activity of long-chain fatty alcohols against Staphylococcus aureus. Molecules. 2007;12:139-48.
14. Aparna V, Dileep KV, Mandal PK, Karthe P, Sadasivan C, Haridas, M. Anti-inflammatory property of n-hexadecanoic acid: structural evidence and kinetic assessment. Chem Biol Drug Des. 2012;80:434-9.
15. Shukla R, Banerjee S, Tripathi YB. Antioxidant and Antiapoptotic effect of aqueous extract of *Pueraria tuberosa* (Roxb. Ex Willd.) DC. On streptozotocin-induced diabetic nephropathy in rats. BMC Complement Altern Med. 2018;18:156.
16. Roy RN, Laskar S, Sen SK. Dibutyl phthalate, the bioactive compound produced by *Streptomyces albidoflavus* 321.2. Microbiol Res. 2006;161:121-6.
17. Thirumalanadhuni V, Vani M, Maheswari D, Yerraguravagari, LL. Insilico Anti-Inflammatory Activity of Dibutylphthalate. IADS International Conference on Computing, Communications & Data Engineering (CCODE) 2018. http://dx.doi.org/10.2139/ssrn.3167809.

18. Chandrasekaran M, Senthilkumar A, Venkatesalu V. Antibacterial and antifungal efficacy of fatty acid methyl esters from the leaves of *Sesuvium portulacastrum* L. Eur Rev Med Pharmacol Sci. 2011;15:775-80.
19. Da Silva AD, Machado AS, Tempête C, Robert-Gero M. Synthesis and biological activity of methyl-D-glucopyranoside derivatives of mercaptopurine and mercaptopyrimidine. Eur J Med Chem. 1994;29:149-52.
20. Begum IF, Mohankumar R, Jeevan M, Ramani K. GC–MS analysis of bio-active molecules derived from *Paracoccus pantotrophus* FMR19 and the antimicrobial activity against bacterial pathogens and MDROs. Indian J Microbiol. 2016;56:426-32.
21. Krishnamoorthy K, Subramaniam P. Phytochemical profiling of leaf, stem, and tuber parts of *Solena amplexicaulis* (Lam.) Gandhi using GC-MS. Int Sch Res Notices. 2014;2014. https://doi.org/10.1155/2014/567409.
22. Yu FR, Lian XZ, Guo HY, McGuire PM, Li RD, Wang R, et al. Isolation and characterization of methyl esters and derivatives from *Euphorbia kansui* (Euphorbiaceae) and their inhibitory effects on the human SGC-7901 cells. J Pharm Pharm Sci. 2005;8:528-35.
23. Mujeeb F, Bajpai P, Pathak N. Phytochemical evaluation, antimicrobial activity, and determination of bioactive components from leaves of *Aegle marmelos*. BioMed Res Int. 2014;2014. https://doi.org/10.1155/2014/497606.
24. Abubakar M, Majinda R. GC-MS analysis and preliminary antimicrobial activity of *Albizia adianthifolia* (Schumach) and *Pterocarpus angolensis* (DC). Medicines. 2016;3:3.
25. Habib MR, Karim MR. Antimicrobial and cytotoxic activity of di-(2-ethylhexyl) phthalate and anhydrosophoradiol-3-acetate isolated from *Calotropis gigantea* (Linn.) flower. Mycobiology. 2009;37:31-6.
26. Cárdeno A, Aparicio-Soto M, Montserrat-de la Paz S, Bermudez B, Muriana FJ, Alarcón-de-la-Lastra C. Squalene targets pro-and anti-inflammatory mediators and pathways to modulate over-activation of neutrophils, monocytes and macrophages. J Funct Foods. 2015;14:779-90.
27. Garcia-Bermudez J, Baudrier L, Bayraktar EC, Shen Y, La K, Guarecuco R, et al. Squalene accumulation in cholesterol auxotrophic lymphomas prevents oxidative cell death. Nature. 2019;567:118.
28. Rao CV, Newmark HL, Reddy BS. Chemopreventive effect of squalene on colon cancer. Carcinogenesis. 1998;19:287-90.
29. Nakagawa M, Yamaguchi T, Fukawa H, Ogata J, Komiyama S, Akiyama SI, et al. Potentiation by squalene of the cytotoxicity of anticancer agents against cultured mammalian cells and murine tumor. Jpn J Cancer Res. 1985;76:315-20.
30. Rameshkumar R, Satish L, Pandian S, Rathinapriya P, Rency AS, Shanmugaraj G, et al. Production of squalene with promising antioxidant properties in callus cultures of *Nilgirianthus ciliatus*. Ind Crops Prod. 2018;126:357-67.
31. Hensley K, Benaksas EJ, Bolli R, Comp P, Grammas P, Hamdheydari L, et al. New perspectives on vitamin E: γ-tocopherol and carboxyethylhydroxychroman metabolites in biology and medicine. Free Radic Biol Med. 2004;36:1-15.
32. Jiang Q, Elson-Schwab I, Courtemanche C, Ames BN. γ-Tocopherol and its major metabolite, in contrast to α-tocopherol, inhibit cyclooxygenase activity in macrophages and epithelial cells. Proc Natl Acad Sci U.S.A. 2000;97:11494-9.
33. Shin J, Yang SJ, Lim Y. Gamma-tocopherol supplementation ameliorated hyper-inflammatory response during the early cutaneous wound healing in alloxan-induced diabetic mice. Exp Biol Med. (Maywood) 2017;242:505-15.
34. Gopalan A, Yu W, Jiang Q, Jang Y, Sanders BG, Kline K. Involvement of de novo ceramide synthesis in gamma-tocopherol and gamma-tocotrienol-induced apoptosis in human breast cancer cells. Mol Nutr Food Res. 2012;6:1803-11.

35. Tahan G, Aytac E, Aytekin H, Gunduz F, Dogusoy G, Aydin S, et al. Vitamin E has a dual effect of anti-inflammatory and antioxidant activities in acetic acid–induced ulcerative colitis in rats. Can J Surg. 2011;54:333.
36. Jiang Q. Natural forms of vitamin E: metabolism, antioxidant, and anti-inflammatory activities and their role in disease prevention and therapy. Free Radic Biol Med. 2014;72:76-90.
37. Wang Y, Park NY, Jang Y, Ma A, Jiang Q. Vitamin E γ-tocotrienol inhibits cytokine-stimulated NF-κB activation by induction of anti-inflammatory A20 via stress adaptive response due to modulation of sphingolipids. J Immunol Res. 2015;126-33. doi: 10.4049/jimmunol.1403149.
38. Ehrlich HP, Tarver H, Hunt TK. Inhibitory effects of vitamin E on collagen synthesis and wound repair. Ann Surg. 1972;175:235.
39. Parihar G, Balekar N. Isolation and characterisation of stigmast-5-en-3-ol (β-sitosterol) from *Calotropis procera* latex ethyl acetate fraction for immunomodulatory activity. Int J Pharm Sci Res. 2008;8:1375-80.
40. Fernando IS, Sanjeewa KA, Ann YS, Ko CI, Lee SH, Lee WW, et al. Apoptotic and antiproliferative effects of Stigmast-5-en-3-ol from *Dendronephthya gigantea* on human leukemia HL-60 and human breast cancer MCF-7 cells. Toxicol In Vitro. 2018;52:297-305.
41. Penduka D, Buwa L, Mayekiso B, Basson AK, Okoh AI. Identification of the antiListerial constituents in partially purified column chromatography fractions of *Garcinia kola* seeds and their interactions with standard antibiotics. Evid Based Complementary Altern Med. 2014;2014. https://doi.org/10.1155/2014/850347.
