# Supplementary material for: Validating potent anti-inflammatory and anti-rheumatoid properties of Drynaria quercifolia rhizome methanolic extract through in vitro, in vivo, in silico and GC-MS-based profiling
Source: BMC Complement Med Ther. 2021 Mar 12;21:89. doi: 10.1186/s12906-021-03265-7 (PMC7953762; doi:10.1186/s12906-021-03265-7)
Supplement: Supplementary file 3 — Additional file 3: Table S1. Molecular docking scores of bioactive compounds identified in Drynaria quercifolia methanolic extracts with COX-2, IL-6, and TNF-α. [file 12906_2021_3265_MOESM3_ESM.pdf]

TITLE

Validating potent anti-inflammatory and anti-rheumatoid properties of *Drynaria quercifolia* rhizome methanolic extract through *in vitro, in vivo, in silico and* GC-MS-based profiling.

Authors:

Debabrata Modak[1], Subhashis Paul[1], Sourav Sarkar[1], Subarna Thakur[2] and Soumen Bhattacharjee[1*].

[1]Cell and Molecular Biology Laboratory, Department of Zoology, University of North Bengal, Darjeeling 734013, West Bengal, India.

[2]Department of Bioinformatics, University of North Bengal, Darjeeling 734013, West Bengal, India.

Title: Additional file 3

Description: Table 1: Molecular docking scores of bioactive compounds identified in *Drynaria quercifolia* methanolic extracts with COX-2, IL-6, and TNF-α.

Protein	Compound	Binding energy (Kcal/Mol)	Inhibition constant in micromolar	No. of H bonds (drug-enzyme)	Amino acid involved in interaction	Other interacting residues
COX-2	Squalene	-9.59	0.094 µM	Nil	Nil	ALA199, ALA202, GLN203, THR 206, HIS207, PHE210, LYS211, THR212, GLN 289, VAL291, ASN 382, TYR385, HIS 386, TRP, 387, HIS 388, LEU 390, LEU 391
	Gamma Tocopherol	-9.06	0.228 µM	1	ALA 199	ALA 202, HIS 207, PHE 210, VAL 291, HIS 386, HIS 388, LEU 390, LEU 391
	Dibutyl Phthalate	-7.14	5.83 µM	2	HIS 207 HIS 388	ALA199, PHE200, ALA 202, GLN 203, THR 5, PHE 210, ASN 382, TYR 385, HIS386, TRP 387, LEU390, LEU 391
	9,12-Octadecadienoic acid (Z,Z)-, methyl ester_Methyl Linoleate	-6.72	11.85 µM	1	ARG 120	VAL116, VAL349, LEU352, SER 353, TYR 355, LEU 359, PHE381, LEU 384, TYR 385, TRP 387, PHE518, MET 522, VAL 523, GLY 526, ALA 527, SER 530, LEU 531
	Vitamin E_ Alpha-Tocopherol	-6.48	17.81 µM	Nil	Nil	HIS90, ARG 120, VAL 349, LEU 352, SER 353, LEU359,PHE 381, LEU384, TYR 385, TRP 387, ARG 513,

						PHE 518, MET 522, VAL 523, GLY 526, ALA527, SER530, LEU 531
	n Hexadecanoic acid	-6.47	17.97 µM	2	ARG 120 TYR 355	TYR348, VAL 349, LEU 352, SER 353, LEU 384, TYR 385, TRP 387, PHE518, MET 522, VAL 523, GLY 526, ALA 527
	Phenylacetic Acid	-5.72	64.12 µM	Nil	Nil	ALA199, ALA 202, GLN 203, THR206, HIS207, PHE210, LYS211, THR212, GLN 289, VAL 291, ASN382, TYR 385, HIS 386, TRP387, HIS388, LEU 390, LEU 391
IL-6	Squalene	-4.60	423.72 µM	Nil	Nil	ARG 30, LEU 33, ILE 36, SER 37, LYS171, GLU 172, GLN 175, SER176, ARG 178, ARG 179, ARG 182
	Gamma Tocopherol	-5.10	183.63 µM	2	GLN175 ARG179	ARG 30, LEU 33, ASP 34, SER 176, LEU 178, ARG 182
	Dibutyl Phthalate	-4.07	1.03 mM	2	ARG179 ARG182	ASP26, ARG 30, GLN175, LEU 178
	9,12-Octadecadienoic acid (Z,Z)-, methyl ester_Methyl Linoleate	-4.15	909.72 µM	2	ARG179 ARG182	ARG30, LEU33, ASP34, SER37, LYS171, GLN175, LEU178
	Vitamin E_ Alpha-Tocopherol	-5.29	132.56 µM	2	GLN175 ARG179	ASP26, ARG 30, LEU33, SER176, LEU 178, ARG182
	n Hexadecanoic acid	-5.01	211.55 µM	2	ARG179 ARG182	ARG30, LEU 33, ASP34, GLN 175, LEU 178
	Phenylacetic Acid	-5.55	85.95 µM	2	ARG179 ARG182	ASP 26, ARG30, GLN175, LEU178

TNF-α	Squalene	-6.70	12.36 µM	Nil	Nil	LEU 57, ILE 58, TYR 59, SER 60, GLN 61, TYR119, LEU 120, GLY 121, GLY 122, TYR 151, ILE 155
	Gamma Tocopherol	-7.44	3.50 µM	1	LEU 120	LEU 57, ILE 58, TYR 59, SER 60, GLN 61, TYR 119, GLY 121, GLY 122, TYR 151, ILE 155
	Dibutyl Phthalate	-6.17	30.20 µM	1	TYR 151	LEU 57, ILE58, TYR 59, SER 60, GLN 61, TYR 119, LEU 120, GLY 121
	9,12-Octadecadienoic acid (Z,Z)-, methyl ester_Methyl Linoleate	-5.22	148.45 µM	2	GLN 175 ARG 179	ASP 26, ARG 30, LEU 33, SER 176, LEU 178, ARG 182
	Vitamin E_ Alpha-Tocopherol	-7.69	2.30 µM	Nil	Nil	LEU 57, ILE 58, TYR 59, SER 60, GLN 61, TYR 119, LEU 120, GLY 121, GLY 122, TYR 151
	n Hexadecanoic acid	-4.77	318.88 µM	1	TYR 151	LEU 57, ILE 58, TYR 59, SER 60, GLN 61, TYR 119, LEU 120, GLY 121
	Phenylacetic Acid	-4.36	635.30 µM	3	LYS 98, PRO 117, TYR 119	GLU 116, ILE 118
