# Supplementary material for: Validating potent anti-inflammatory and anti-rheumatoid properties of Drynaria quercifolia rhizome methanolic extract through in vitro, in vivo, in silico and GC-MS-based profiling
Source: BMC Complement Med Ther. 2021 Mar 12;21:89. doi: 10.1186/s12906-021-03265-7 (PMC7953762; doi:10.1186/s12906-021-03265-7)
Supplement: Supplementary file 4 — Additional file 4 Figure S1. Represents the interaction between the COX-2 and the inhibitor, Squalene. Figure 2. Represents the interaction between the COX-2 and the inhibitor, Dibutyl phthalate. Figure 3. Represents the interaction between the COX-2 and the inhibitor, 9,12-Octadecadienoic acid (Z,Z)-, methyl ester_Methyl Linoleate. Figure 4. Represents the interaction between the COX-2 and the inhibitor, Vitamin E, Alpha –Tocopherol. Figure 5. Represents the interaction between the COX-2 and the inhibitor, n Hexadecanoic acid. Figure 6. represents the interaction between the COX-2 and the inhibitor, Phenylacetic Acid. Figure 7. represents the interaction between the TNF-α and the inhibitor, Squalene. Figure 8. Represents the interaction between the TNF-α and the inhibitor, Dibutyl phthalate. Figure 9. Represents the interaction between the TNF-α and the inhibitor, 9,12-Octadecadienoic acid (Z,Z)-, methyl ester_Methyl Linoleate. Figure 10. Represents the interaction between the TNF-α and the inhibitor, Vitamin E, Alpha –Tocopherol. Figure 11. Represents the interaction between the TNF-α and the inhibitor, n Hexadecanoic acid. Figure 12. Represents the interaction between the TNF-α and the inhibitor, n Phenylacetic Acid. Figure 13. Represents the interaction between the IL-6 and the inhibitor, Squalene. Figure 14. Represents the interaction between the IL-6 and the inhibitor, Dibutyl Phthalate. Figure 15. Represents the interaction between the IL-6 and the inhibitor, 9,12-Octadecadienoic acid (Z,Z)-, methyl ester_Methyl Linoleate. Figure 16. Represents the interaction between the IL-6 and the inhibitor, Vitamin E, Alpha –Tocopherol. Figure 17. Represents the interaction between the IL-6 and the inhibitor, n Hexadecanoic acid. Figure 18. Represents the interaction between the IL-6 and the inhibitor, Phenylacetic Acid. [file 12906_2021_3265_MOESM4_ESM.pdf]

TITLE

Validating potent anti-inflammatory and anti-rheumatoid properties of *Drynaria quercifolia* rhizome methanolic extract through *in vitro, in vivo, in silico and* GC-MS-based profiling.

Authors:

Debabrata Modak[1], Subhashis Paul[1], Sourav Sarkar[1], Subarna Thakur[2] and Soumen Bhattacharjee[1*].

[1]Cell and Molecular Biology Laboratory, Department of Zoology, University of North Bengal, Darjeeling 734013, West Bengal, India.

[2]Department of Bioinformatics, University of North Bengal, Darjeeling 734013, West Bengal, India.

<u>Title</u>: Additional file 4

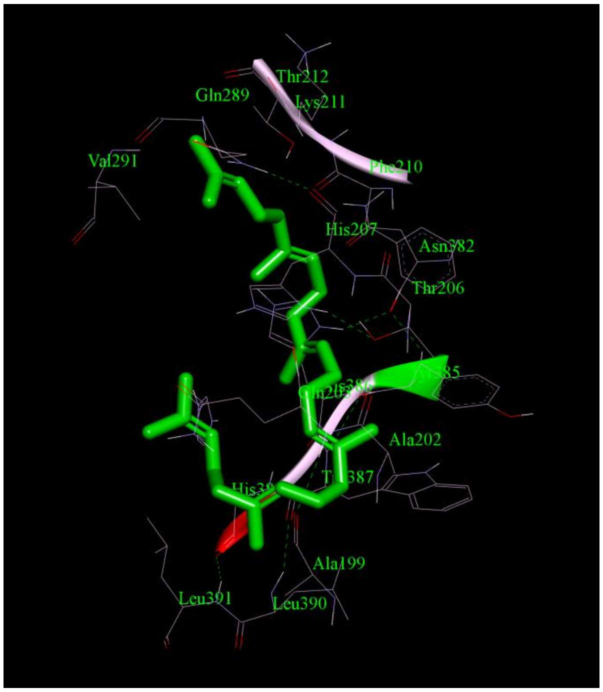

<u>Description</u>: Figure 1 represents the interaction between the COX-2 and the inhibitor, Squalene.

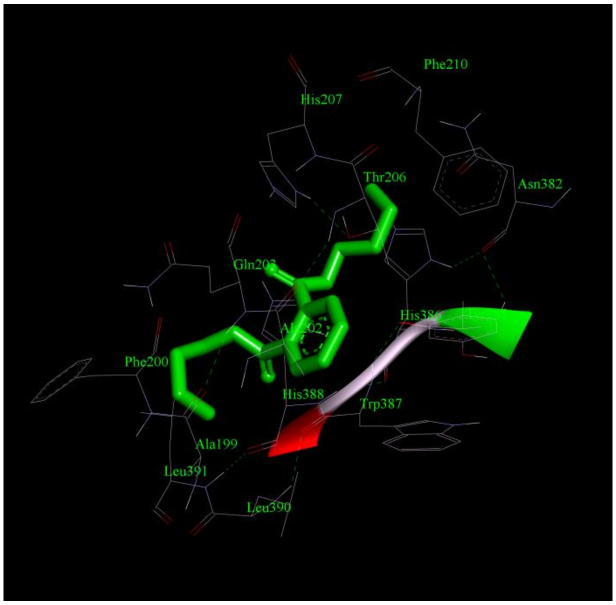

<u>Description</u>: Figure 2 represents the interaction between the COX-2 and the inhibitor, Dibutyl phthalate.

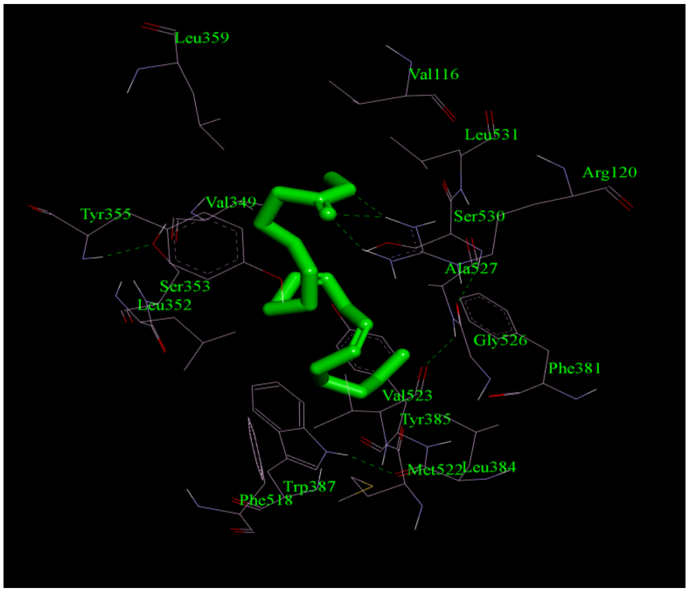

<u>Description</u>: Figure 3 represents the interaction between the COX-2 and the inhibitor, 9,12-Octadecadienoic acid (Z,Z)-, methyl ester_Methyl Linoleate.

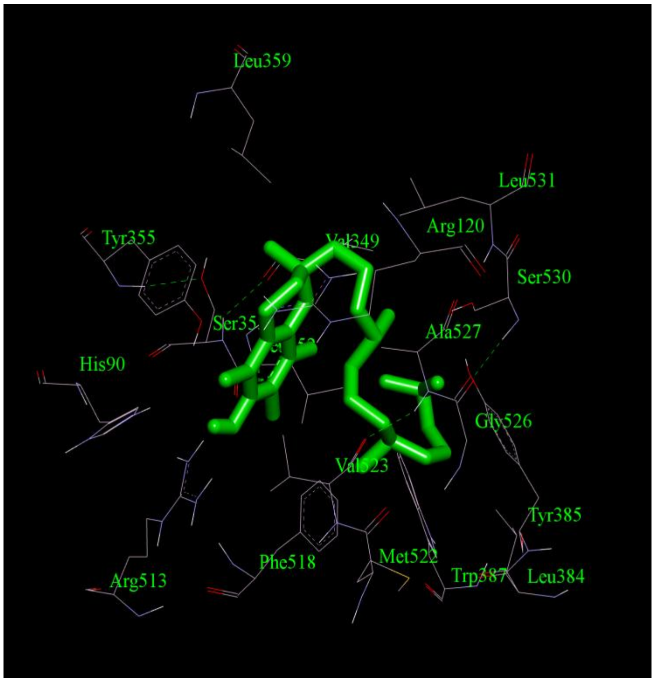

<u>Description</u>: Figure 4 represents the interaction between the COX-2 and the inhibitor, Vitamin E, Alpha –Tocopherol.

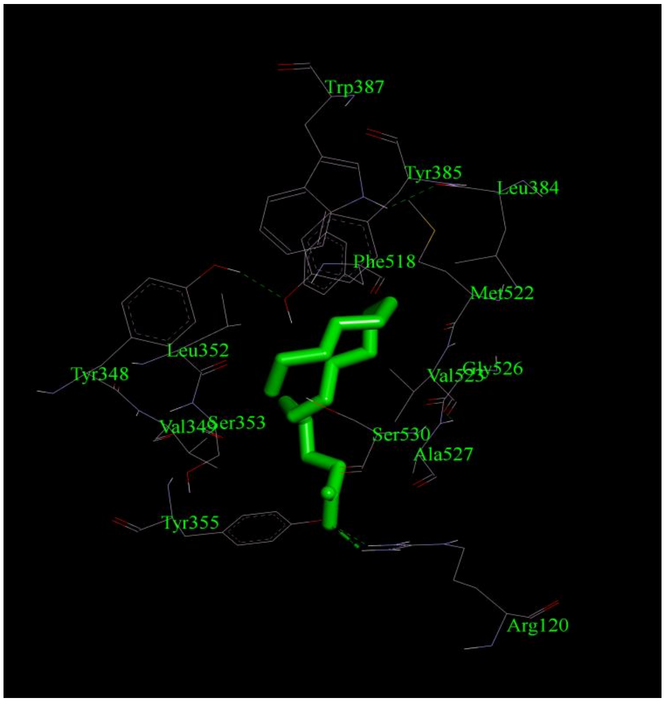

<u>Description</u>: Figure 5 represents the interaction between the COX-2 and the inhibitor, n Hexadecanoic acid.

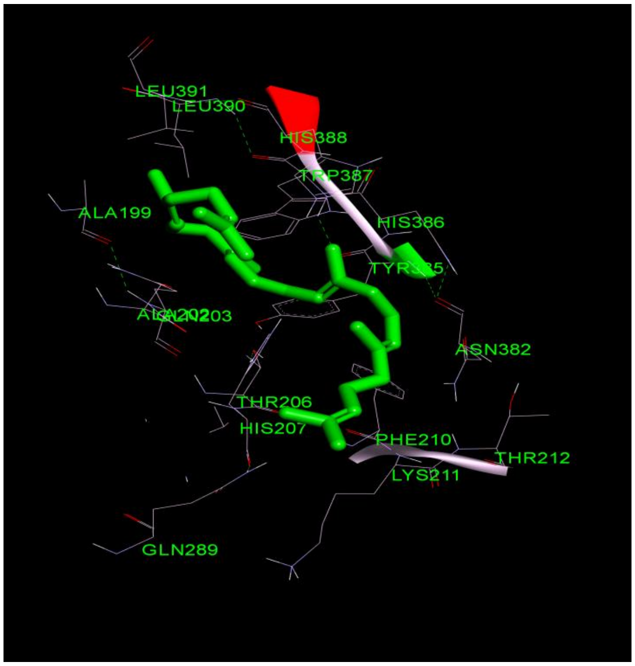

<u>Description</u>: Figure 6 represents the interaction between the COX-2 and the inhibitor, Phenylacetic Acid.

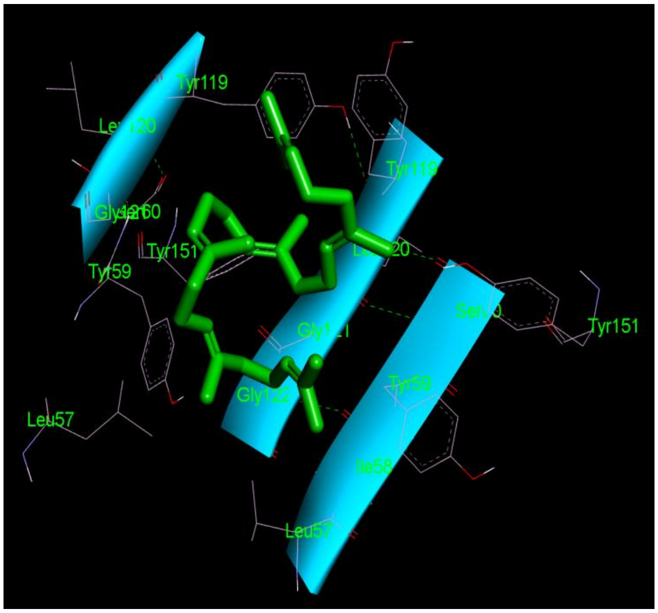

<u>Description</u>: Figure 7 represents the interaction between the TNF-α and the inhibitor, Squalene.

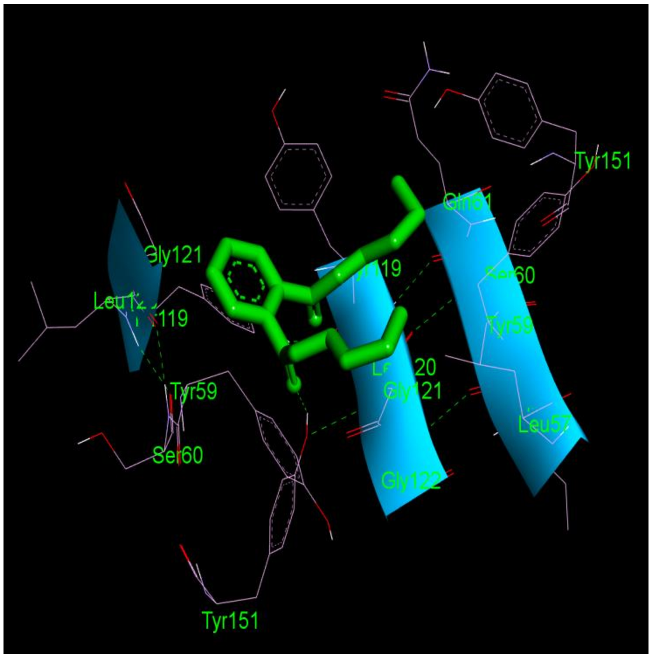

<u>Description</u>: Figure 8 represents the interaction between the TNF-α and the inhibitor, Dibutyl phthalate.

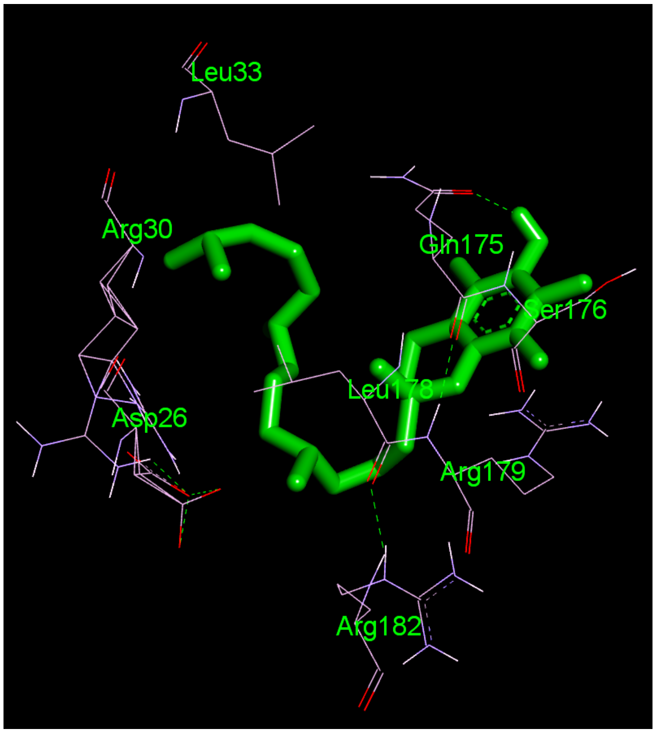

<u>Description</u>: Figure 9 represents the interaction between the TNF-α and the inhibitor, 9,12-Octadecadienoic acid (Z,Z)-, methyl ester_Methyl Linoleate.

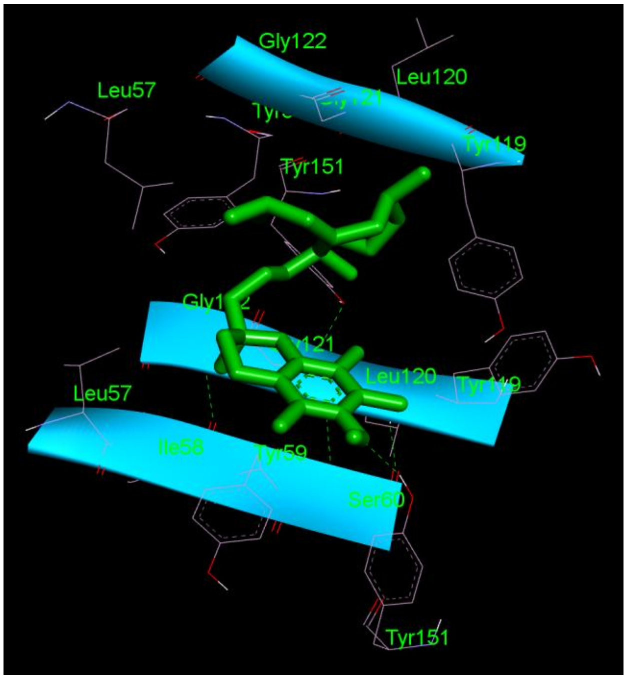

<u>Description</u>: Figure 10 represents the interaction between the TNF-α and the inhibitor, Vitamin E, Alpha –Tocopherol.

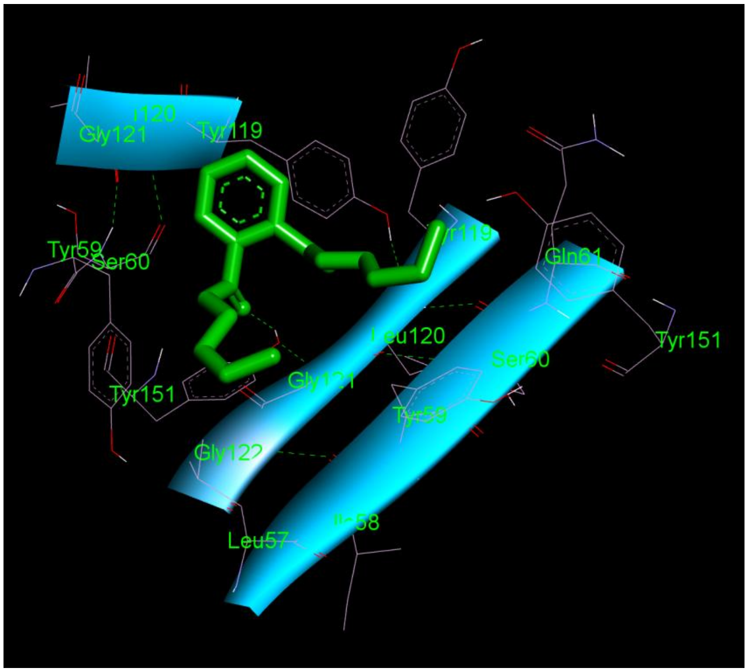

<u>Description</u>: Figure 11 represents the interaction between the TNF-α and the inhibitor, n Hexadecanoic acid.

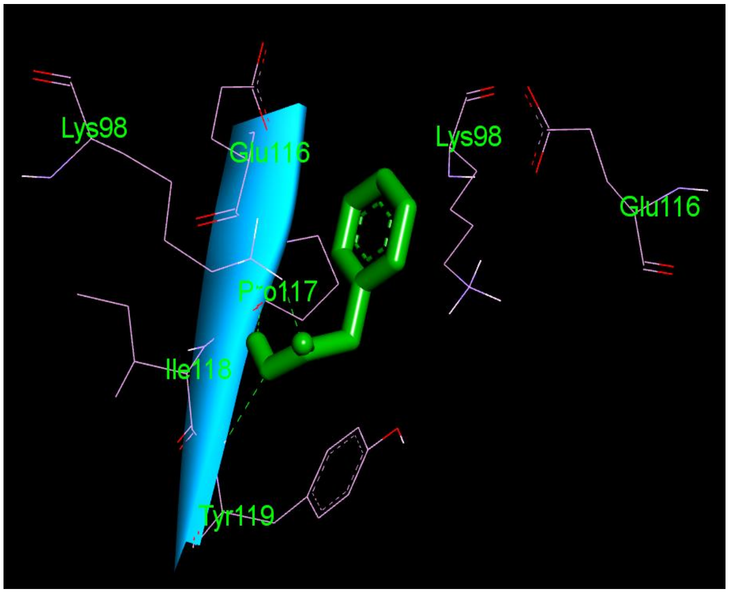

<u>Description</u>: Figure 12 represents the interaction between the TNF-α and the inhibitor, n Phenylacetic Acid.

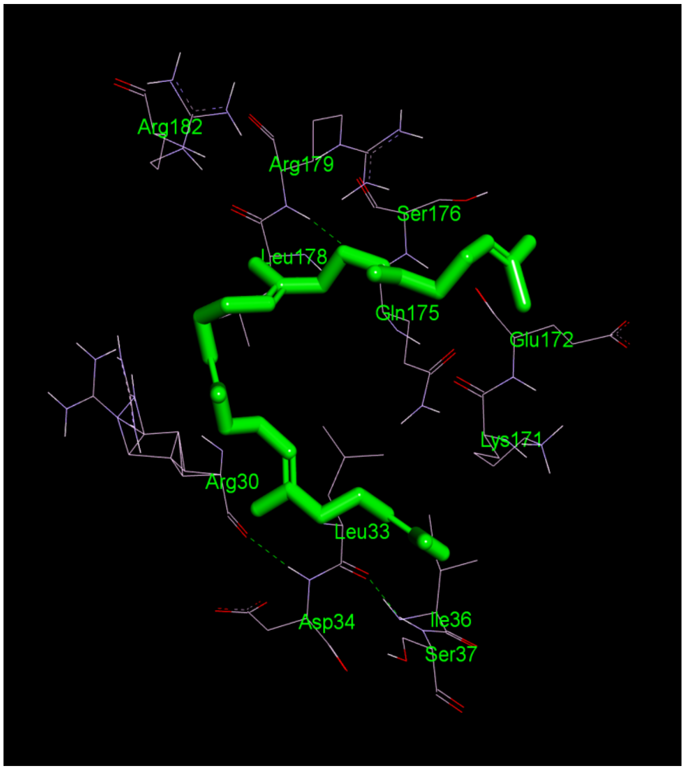

Description: **Figure 13 represents the interaction between the IL-6 and the inhibitor, Squalene.**

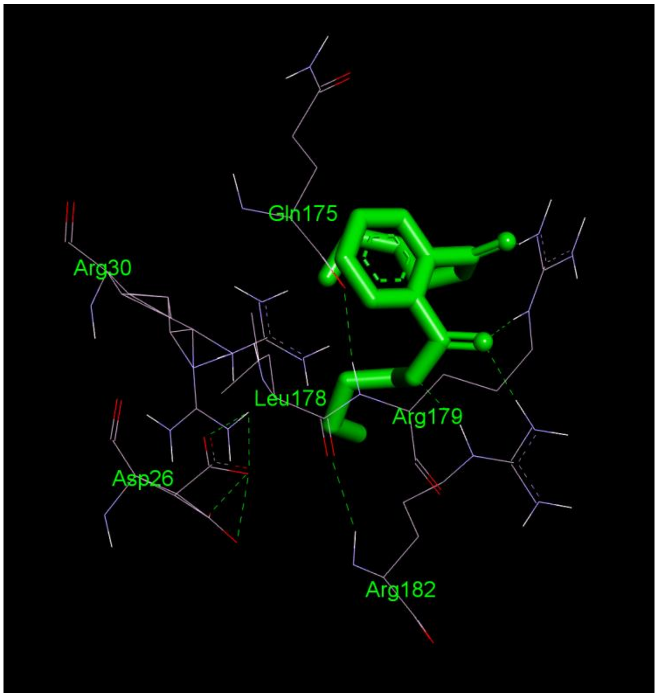

Description: **Figure 14 represents the interaction between the IL-6 and the inhibitor, Dibutyl Phthalate.**

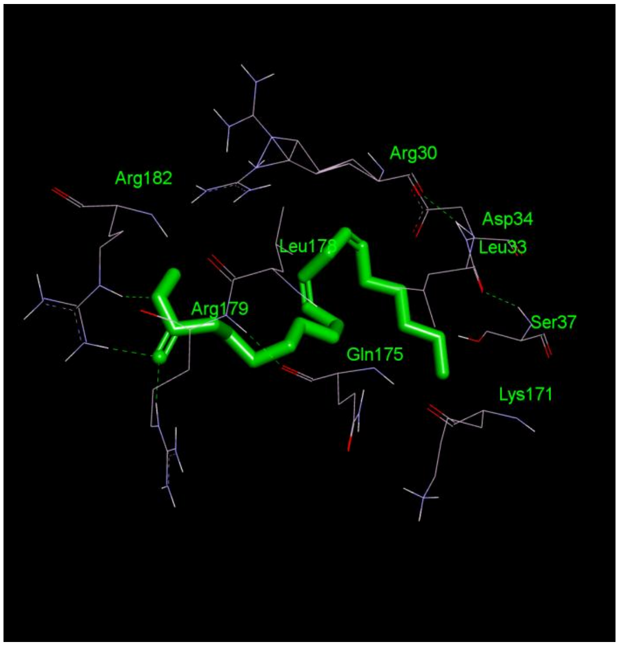

<u>Description</u>: Figure 15 represents the interaction between the IL-6 and the inhibitor, 9,12-Octadecadienoic acid (Z,Z)-, methyl ester_Methyl Linoleate.

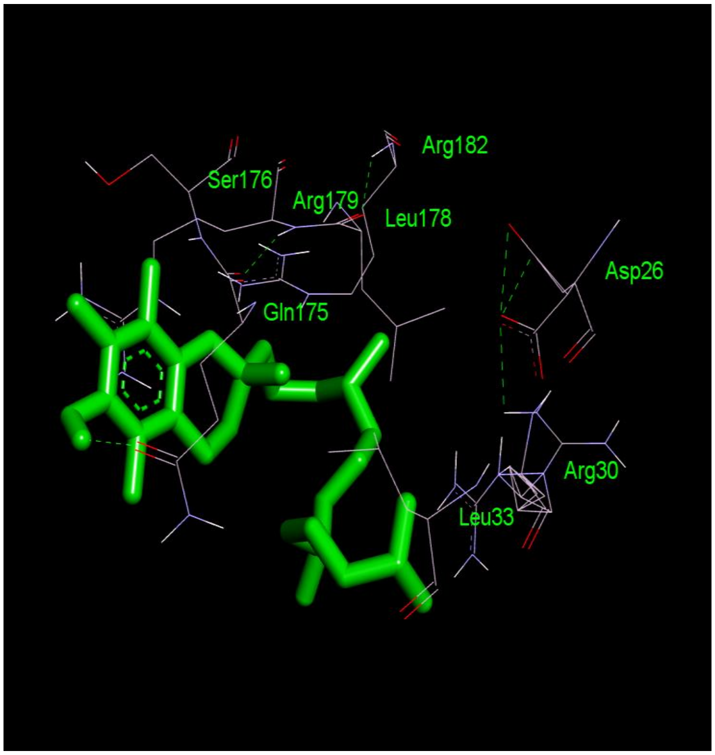

<u>Description</u>: Figure 16 represents the interaction between the IL-6 and the inhibitor, Vitamin E, Alpha –Tocopherol.

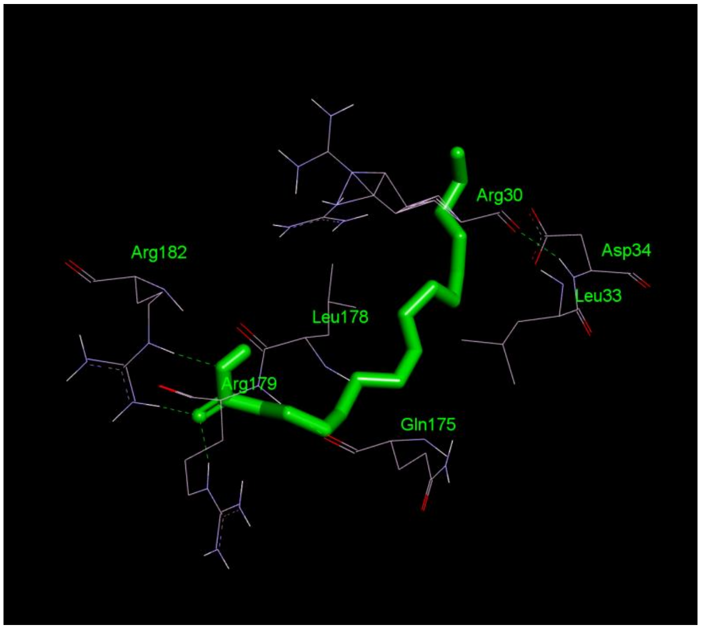

Description: **Figure 17 represents the interaction between the IL-6 and the inhibitor, n Hexadecanoic acid.**

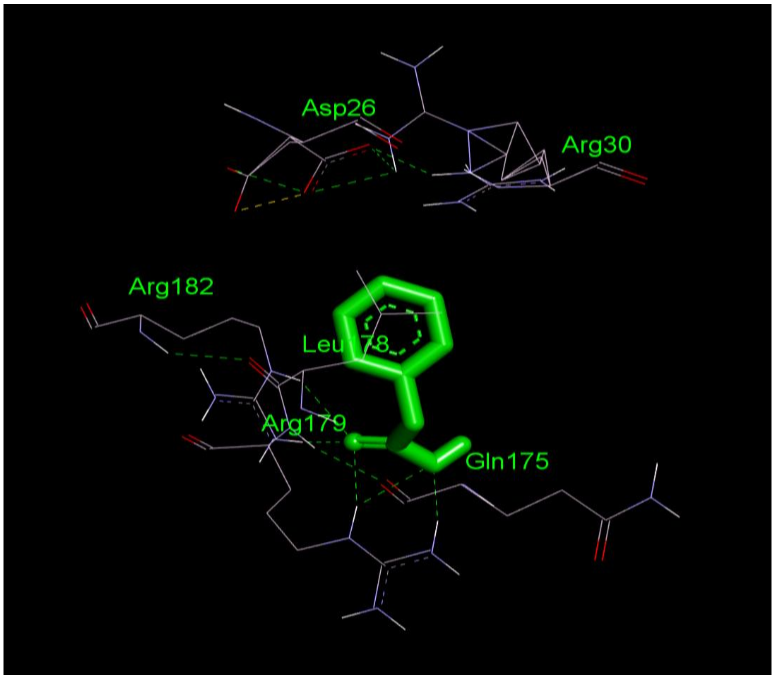

Description: **Figure 18 represents the interaction between the IL-6 and the inhibitor, Phenylacetic Acid.**
